# The use of isoxazoline and isoxazole scaffolding in the design of novel thiourea and amide liquid-crystalline compounds

**DOI:** 10.3762/bjoc.16.20

**Published:** 2020-02-06

**Authors:** Itamar L Gonçalves, Rafaela R da Rosa, Vera L Eifler-Lima, Aloir A Merlo

**Affiliations:** 1 Laboratório de Síntese Orgânica Medicinal/LaSOM, Faculdade de Farmácia, Universidade Federal do Rio Grande do Sul, Avenida Ipiranga, Porto Alegre/RS, Brazil; 2 CENIMAT/i3N, Departamento de Ciência dos Materiais, Faculdade de Ciências e Tecnologia, Universidade NOVA de Lisboa, 2829-516 Caparica, Portugal; 3 Institute of Chemistry, Universidade Federal do Rio Grande do Sul, Porto Alegre, RS, Brazil

**Keywords:** amides, isoxazole, isoxazoline, liquid crystal, thiourea

## Abstract

A series of novel thiourea and amide liquid crystals containing 5-membered isoxazoline and isoxazole rings were synthetized and the liquid crystal properties studied. Thioureas were obtained using a condensation reaction of benzoyl chlorides, arylamines and ammonium thiocyanate. The amides, on the other hand, were the byproduct of a quantitative reaction which used potassium cyanate as the starting material. Thiourea and amide derivatives were predominantly SmA mesophase inductors. A nematic mesophase was observed only for thioureas and amides containing an isoxazole ring. Additionaly, the liquid crystal behavior was also dependent on the relative position of nitrogen and oxygen atoms on the 5-membered heterocycle.

## Introduction

Thioureas are a structurally diversified group of organic compounds, with technological applications in different areas. The structural diversity of the thiourea moiety is linked to possibly one or both nitrogen components, which may be substituted symmetrically or unsymmetrically [1]. Applications of this scaffold include complexing agents in anion sensors [2] organocatalysts [3], intermediates in heterocycle synthesis [1] and development of compounds with pharmacological effects [4].

Considering the importance of this group of molecules, numerous strategies have been developed for their preparation. Substituted thioureas can be obtained using a variety of approaches although the simplest route involves the heating of ammonium thiocyanate with an amine in an aqueous acid medium [5]. They can also be synthesized in the reaction of thiourea with primary amines [6]. Another important synthetic route for substituted thioureas is a two-step approach: (i) the addition/elimination reaction of benzoyl chloride, with a thiocyanate salt, generating in situ, benzoyl isothiocyanate, and (ii) an appropriate amine reaction with benzoyl isothiocyanate, yielding the acylthiourea which may be the final product or may be hydrolyzed to obtain the monosubstituted thiourea [7–8]. An alternative and versatile route for generating isothiocyanate for the use in the preparation of disubstituted symmetrical and unsymmetrical thioureas is the reaction of carbon disulfide with either one or two different amines [9].

Due to their self-assembly and self-organization through intermolecular hydrogen bonding, thioureas display interesting technological applications to this group of molecules, one of which explores its application in liquid crystal design [10–11]. *N,N*′-Bis(3,4,5-trialkoxylphenyl)ureas were identified as columnar liquid crystalline compounds presenting ferroelectric properties [12]. Another study reported cyclic disubstituted ureas with liquid crystalline ferroelectric and antiferroelectric phases [13]. Relating to the thiourea moiety, there is only one recent report, in which some derivatives of *N*-benzoyl-*N*’-arylthiourea with liquid crystalline properties have been investigated [14].

One aspect that compounds forming hydrogen bonds, such as ureas, thioureas, amides etc. for the use in electronic devices is the ability to establish intra- and intermolecular hydrogen bonds [11]. In addition to π–π, dipole–dipole and van der Waals interactions, hydrogen bond interactions are involved in gel formation through self-aggregation of the small gelator molecules. In recent years, there has been increased interest in the study of gels derived from low molecular mass gelators [15–16], and consequently the investigation of gelators of low molar mass with liquid crystal proprieties remains active [17–18].

In addition, isoxazolines and isoxazoles have been identified as promising templates in liquid crystal development, mainly due to their structural and electronic features. Isoxazoline and isoxazole cores show strong dipole moments, polarizabilities, anisotropic interactions and geometrical aspects that favor the formation of stable smectic A and nematic mesophases, respectively [19–20]. This work aimed at synthetizing new thioureas and amides by the installation of mesogenic cores derived from isoxazoline and isoxazole scaffolds and 4-*n*-alkoxybenzoic acid with liquid crystal properties. The liquid crystal properties of the compounds were investigated by polarized optical microscopy analysis (POM) and differential scanning calorimetry (DSC).

## Results and Discussion

### Synthesis

**Scheme 1:**
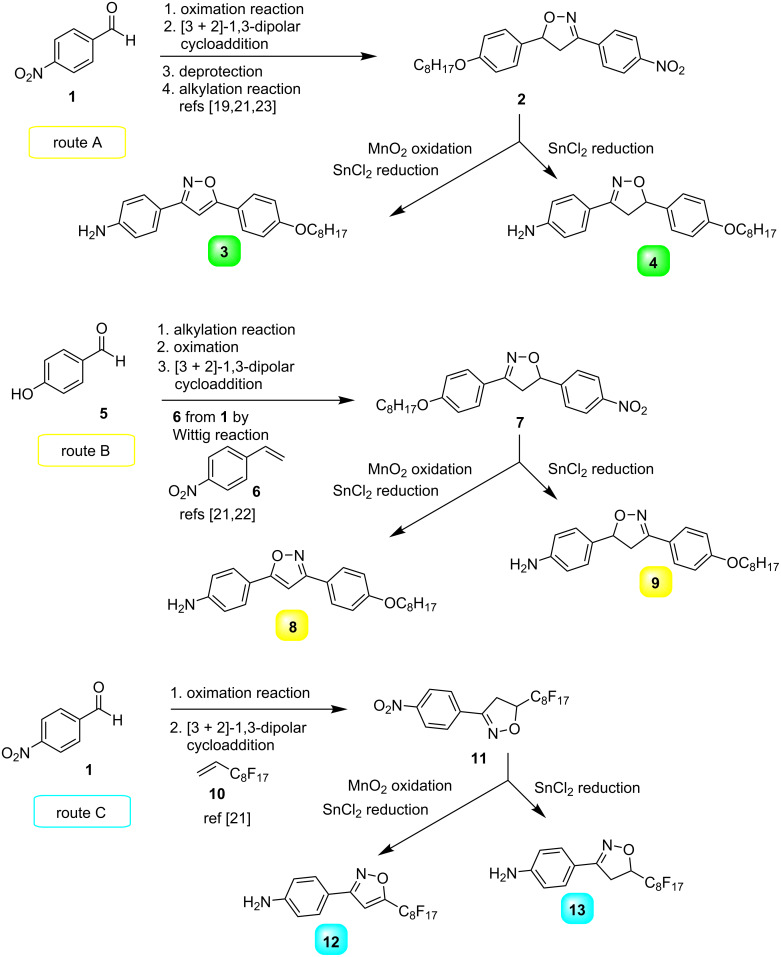
Amines **3**, **4**, **8, 9**, **12** and **13** installed on 5-membered isoxazoline and isoxazole rings.

**Scheme 2:**
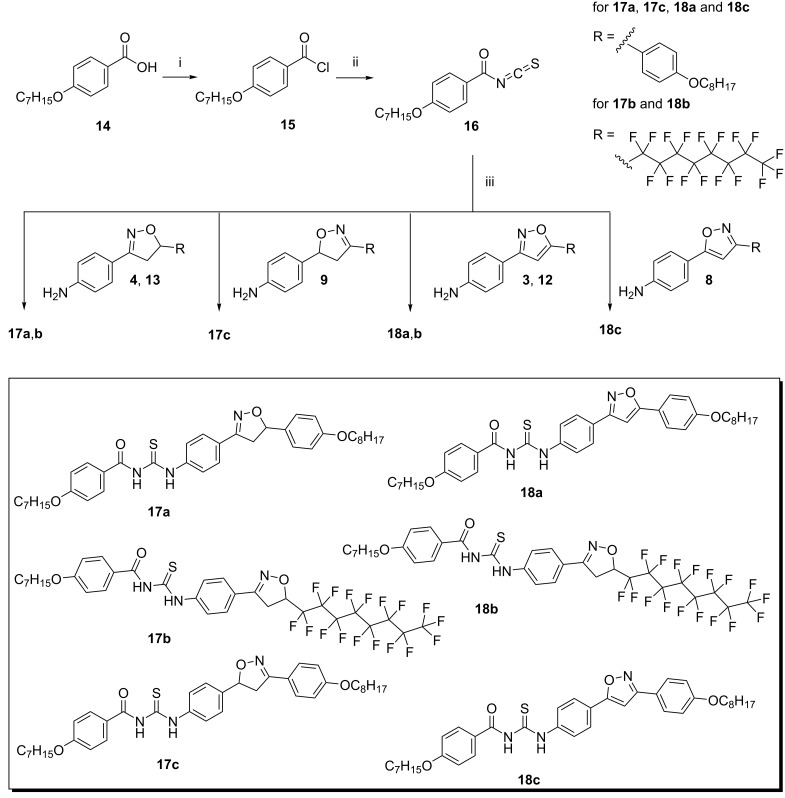
Synthesis of acylisoxazolinylthioureas **17a**–**c** and acylisoxazolylthioureas **18a**–**c**. (i) SOCl_2_, reflux, 2 h; (ii) NH_4_SCN, acetone, reflux 40 minutes; (iii) acetone, reflux.

In order to generate molecular structural diversity in thioureas, three sets of amines carrying 5-membered isoxazolines/isoxazoles (see Scheme 1) were employed and condensed with acyl isothiocyanates, as outlined in Scheme 2. Amines **3**, **4**, **8**, **9**, **12** and **13** were then prepared by [3 + 2]-1,3-dipolar cycloaddition of nitrile oxide (ArCNO) formed in situ from oximes and a number of alkenes as dipolarophiles, to yield the precursors of the cycloadducts isoxazolines **2**, **7** and **11** regioselectively. The cycloadducts were subjected to SnCl_2_ reduction to yield amines **4**, **9** and **13** containing an isoxazoline ring. The amines **3**, **8** and **12** containing an isoxazole ring were synthesized in two sequential steps – first a MnO_2_ oxidation step transforming the isoxazoline ring to an isoxazole ring followed by SnCl_2_ reduction, according to protocols previously published [19,21–24].

The synthetic route used for the synthesis of the hybrid compounds, *N*-acyl-*N*'-isoxazolinylthioureas **17a**–**c** and *N*-acyl-*N*'- isoxazolylthioureas **18a**–**c** is outlined in Scheme 2. The synthesis of thioureas **17a**–**c** and **18a**–**c** involved the activation of 4-heptyloxybenzoic acid (**14**) to its chloride **15**, which was submitted to an acyl nucleophile substitution reaction using ammonium thiocyanate as the nucleophile. This yielded the corresponding acyl isothiocyanate **16**. The solid residue formed was used directly without further isolation and purification. The additional reaction between the acyl isothiocyanate **16** and amines **3**, **4**, **8**, **9**, **12** and **13** yielded the title compounds **17a**–**c** and **18a**–**c** in 56–62% yields (**17a** 57%; **17b** 56%; **17c** 58%; **18a** 61%; **18b** 60%; **18c** 62%).

**Scheme 3:**
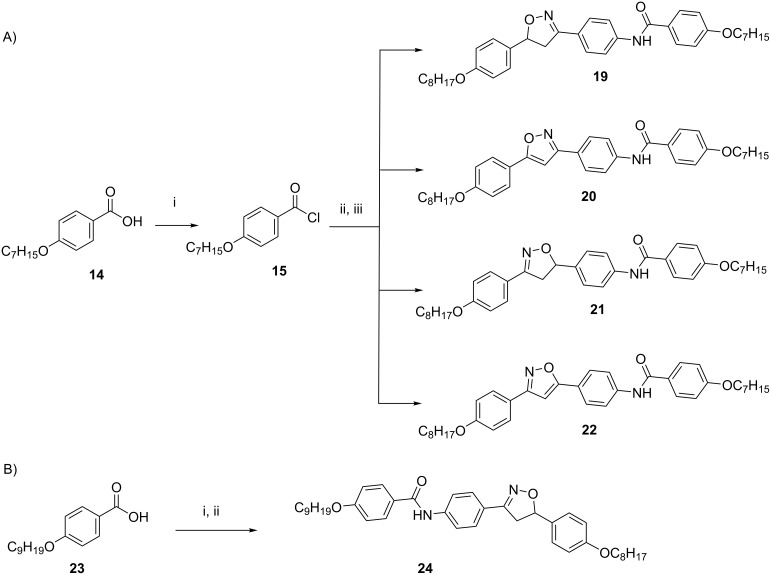
Synthesis of amides. Part A: (i) SOCl_2_, reflux; (ii) KOCN, acetone, reflux; (iii) amines **3, 4**, **8** and **9**, acetone, reflux. Part B: (i) SOCl_2_, reflux; (ii) amine **4**, acetone, reflux.

In an attempt to improve the structural diversity of thioureas, potassium cyanate was used as a nucleophile instead of the thiocyanate salt, following the same experimental protocol. However, when a cyanate salt was used, only the amides **19**–**22** were isolated as the main products. The target ureas were not obtained, according to Scheme 3, part A. To test the hypothesis of intermediate formation of acyl cation [ArCO]^+^ during the condensation reaction of thiocyanate salt, a reaction test was performed to confirm the reactivity between the amines and acyl chloride. This involved the formation and isolation of amide **24**, as a result of the reaction between the reactive intermediate nonyloxybenzoyl chloride from acid **23** and the amine **4** (Scheme 3, part B). Attempts to improve the reactivity of potassium cyanate by adding crown ethers did not result in the formation of ureas as expected. Thus, five new amides **19**–**22** and **24** were obtained in yield range of 42–71% (**19** 42%; **20** 53%; **21** 58%; **22** 53%; **24** 71%).

### Mesomorphic properties

Mesomorphic properties of thioureas **17a**–**c**, and **18a**–**c** and amides **19**–**22** and **24** were investigated by POM and DSC. The DSC results for all compounds are summarized in Table 1.

**Table 1:** Phase transition temperatures (°C), enthalpy/entropy for thioureas **17a**–**c**, **18a**–**c** and amides **19**–**22** and **24** upon heating.^a^

LC	Cr		SmC		SmA		N		I

**17a**	●	157^b^ (6.57/15.27)		–	●	{146}		–	●
**17b**	●	154 (5.66/13.26)		–	●	234^c^		–	●
**17c**	●	116^b,d^ (2.21/5.57)		–		–		–	●
**18a**	●	134 (7.15/17.63)		–		–	●	211 (0.37/0.76)	●
**18b**	●	134 (5.72/14.05)		–	●	217^c^		–	●
**18c**	●	141^e^ (9.23/22.26)		–	●	201^f^	●	202^f^ (0.07/0.14)	●
**19**	●	201 (6.83/14.42)	●	{198}	●	207 (5.14/10.68)		–	●
**20**	●	202 (5.98/12.58)		–	●	271 (0.59/1.09)	●	278 (0.30/0.55)	●
**21**	●	197^d^ (13.53/28.85)		–		–		–	●
**22**	●	208 (6.85/14.24)		–	●	>250		–	●
**24**	●	192^b^ (4.35/9.35)		–	●	206 (9.17/19.12)		–	●

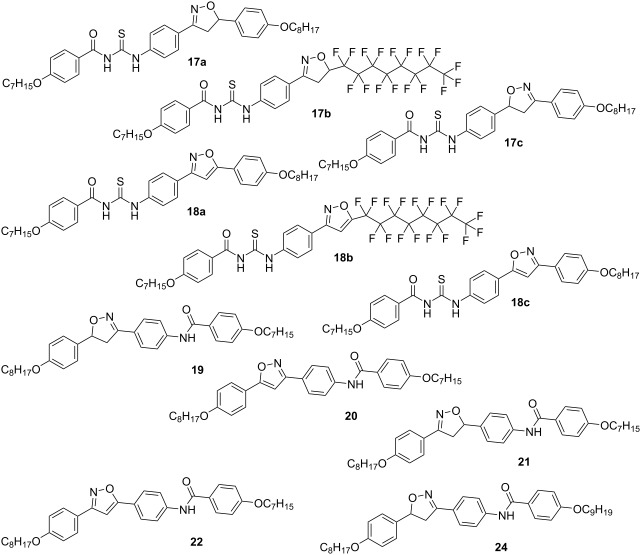									

^a^Data was recorded from the first curve upon heating. Scan 10 °C/min. Cr = crystal phase; SmC = smectic C mesophase; SmA = smectic A mesophase; N = nematic mesophase and I = isotropic phase. Enthalpy and entropy values are given between parentheses in kcal mol^−1^ and cal mol^−1^ K^−1^, respectively. The curly brackets { } represent a monotropic transition temperature. ^b^Other peaks are seen in DSC thermograms below this temperature associated with the thermal history of the sample. ^c^Decomposition was observed when the sample reached this temperature. ^d^No mesophase was detected. ^e^Unresolved shoulder peak at the left side which may be linked to another crystal–crystal transition. ^f^Thermal decomposition started above 200 °C and nematic mesophase coexisted with the isotropic phase until 219 °C.

Transition temperatures, as well as the enthalpy and entropy values of each compound were acquired by differential scanning calorimetry (DSC). The transition temperature peak was considered for all compounds and tabulated, using the first heating curve, as well the values for Δ*H* and Δ*S* associated with each transition (see Table 1). Six liquid crystallines (LCs) thioureas were synthesized and studied and divided into two groups containing isoxazoline rings **17a**–**c** and isoxazoles **18a**–**c**, as well as LCs amides **19**–**22** and **24**. All compounds listed in Table 1 are capable of self-organization by hydrogen bonds, either in their solid state or in solution. Solubility in protic or aprotic polar solvent is restricted at room temperature. However, they become almost soluble in a hot solution of polar solvents, and when cooled, precipitate into an amorphous solid, limiting their use as gelators.

A mesomorphic behavior was observed for thioureas **17a**–**b**, **18a**–**c** and amides **19**, **20**, **22** and **24**. Thiourea **17c** and amide **21** did not display mesomorphic behavior. A smectic A mesophase (SmA) was preponderant in this study. However, a nematic mesophase (N) appeared for thioureas **18a** and **18c** and amide **20**. Thioureas **17b**, **18b** and **18c** and amides **19**, **20, 22** and **24** displayed an enantiotropic SmA mesophase, except thiourea **17a** which showed a monotropic SmA mesophase. A monotropic smectic C mesophase (SmC) at 198 °C was observed for amide **19**. Perfluorinated thioureas **17b** and **18b** presented an SmA mesophase, independent of the nature of the 5-membered heterocycle. The perfluorinated alkyl chain in both compounds is the reason of preference for a lamellar SmA behavior [25–26] by segregation effects. Interestingly, isoxazoline **17a** presented a monotropic SmA mesophase, while its regioisomer **17c** did not show a mesophase. Corresponding isoxazoles formed stable mesophases as expected (N for **18a**; SmA and N for **18c**) [20].

**Figure 1:**
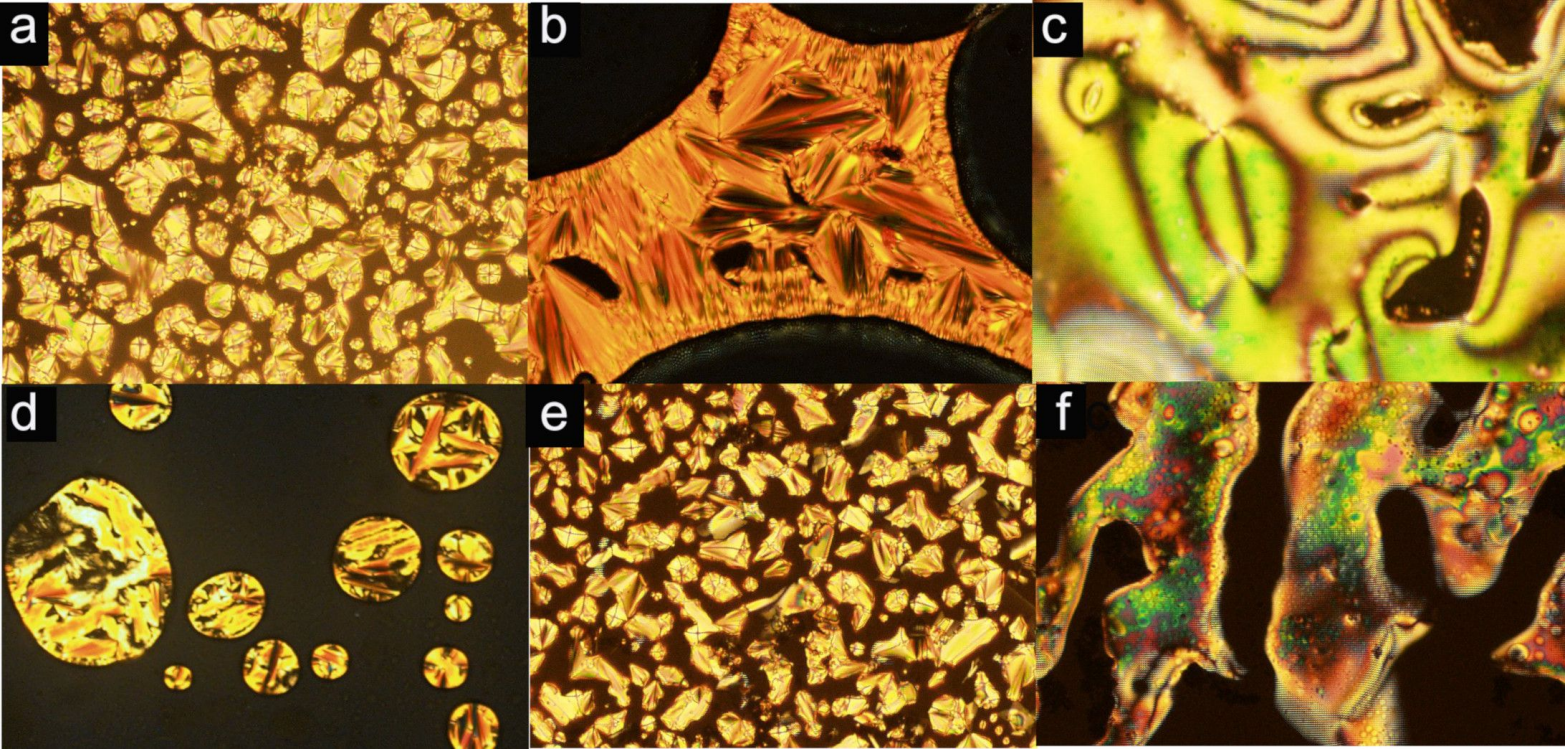
Optical textures observed on POM for thioureas **17a** (a), **17b** (b), **18a** (c), **18b** (d) and **18c** (e,f). All images were obtained upon cooling from the isotropic phase with the exception of (b). The “grid aspect” found in images (c) and (f) is an artifact due to the fast movement of the fluid mesophase.

Figure 1 shows the selected textures of the thiourea series observed under POM. Upon cooling, the fan-shaped focal conic texture of the SmA mesophase was formed for **17a** (Figure 1a), **17b** (Figure 1b), **18b** (Figure 1d) and **18c** (Figure 1e). A nematic mesophase (N) was assigned for thioureas **18a** (Figure 1c) and **18c** by the schlieren texture described in Figure 1f.

**Figure 2:**
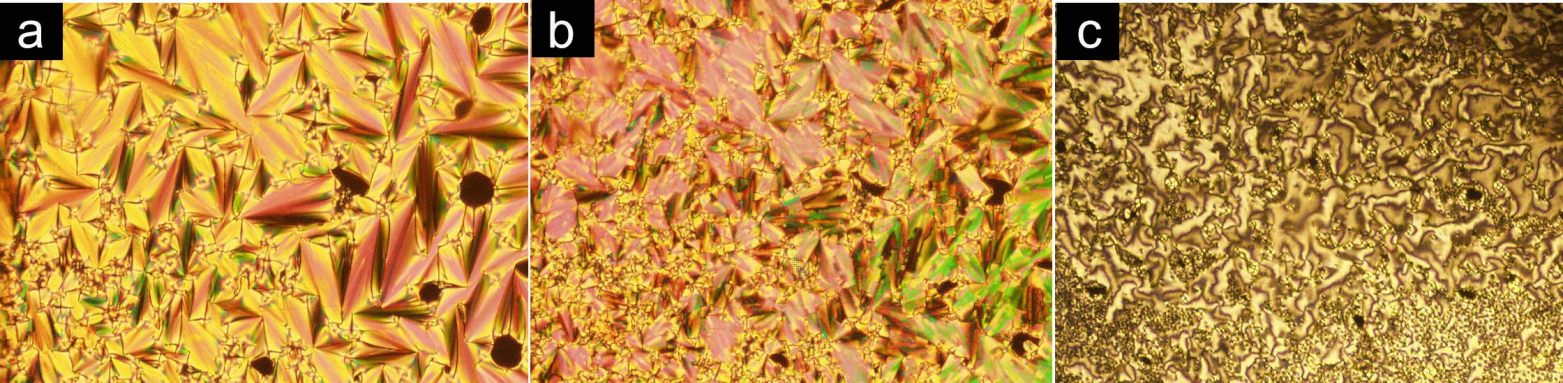
Optical textures observed on POM of amide **19**. (a) Fan-shaped focal conic texture of the SmA mesophase upon cooling at 201 °C; (b) and (c) broken fan-shaped focal conic and schlieren textures of the SmC mesophase upon cooling at 198 °C.

Figure 2 displays the textures recorded for amide **19**. Upon cooling, amide **19** enters the SmA mesophase as evidenced by the fan-shaped focal conic texture (Figure 2a) at 201 °C. At 198 °C, the texture of the sample under analysis becomes blurred. This is indicative of a broken fan-shaped focal conic SmC texture (Figure 2b). Visual inspection in other domains at the same temperature revealed the existence of a schlieren texture (Figure 2c) which corresponds to the monotropic SmC mesophase assignment for amide **19**.

The liquid crystal data tabulated in Table 1 allow to correlate structures and properties of the thioureas and amides. The first comments will be addressed to general aspects of thiourea DSC thermograms. Upon heating, for the first time, peaks can be clearly identified, associated with transition between phases and the thermal history of the samples. Unfortunately, upon cooling, intensity, resolution and peaks of some phase transitions are lost. Considering that molecules self-organize upon cooling, first by hydrogen bonds followed by π-stacking and van der Waals forces, DSC scanning at 10 °C/min is too fast to allow the molecules to self-assemble in a perfect 3D matrix. Even with a scan rate of more than 10 °C, it was not possible to reproduce the peaks reported during the first scan.

**Figure 3:**
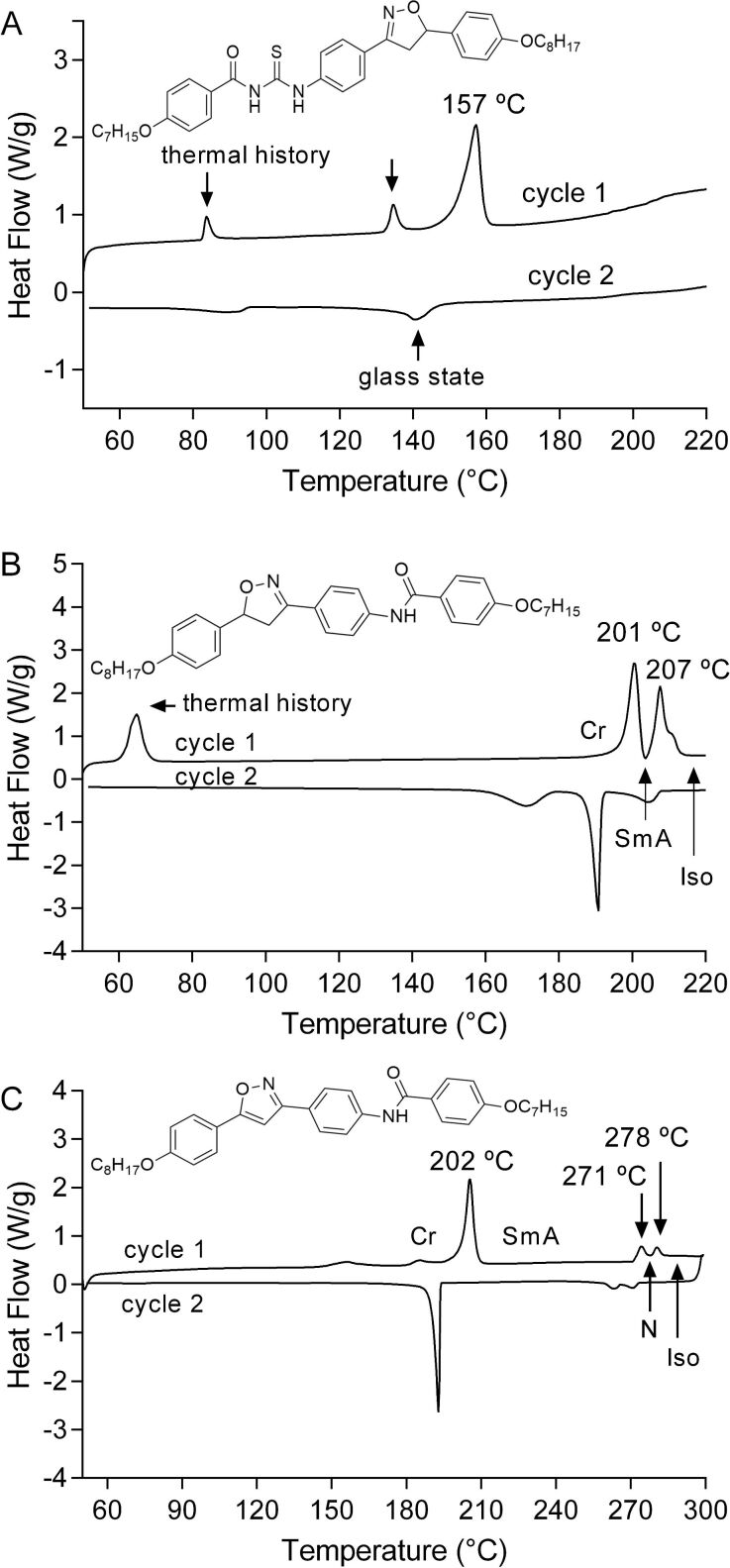
DSC curves for the thiourea **17a** (A), amide **19** (B) and **20** (C) upon the first heating and cooling curve at a rate of 10 °C·min^−1^.

The thermal behavior of the thioureas in this study is similar to that of polymers becoming semicrystalline or an amorphous solid after subjecting them to repeated heating and cooling cycles. Figure 3a is illustrative of the thermal behavior mentioned above for thiourea **17a**. Upon cooling, peaks tend to be less intense and flatter. The DSC thermograms for amides such as **19**, during periods of heating and cooling, display peaks which correspond to the transition between crystal and mesomorphic phases. Figure 3b and Figure 3c describe DSC traces for first heating and cooling curves at 10 °C/min of amides **19** and **20**. Some peaks observed during the first heating cycle are not seen anymore in the second or third heating/cooling cycles due to the thermal history of the samples. Additional DSC graphs may be accessed in Supporting Information File 1.

Thioureas containing isoxazoline rings **17a**–**c** are not prone to show a mesophase, except when they have some special feature that intensifies the segregate character at molecular level [22]. Thus, while **17a** showed a monotropic SmA mesophase, **17b** with a perfluorinated alkyl chain, displayed enantiotropic SmA mesophase with a mesophase range of Δ*T* = 80 °C. Perfluorinated and hydrogenated alkyl chains linked to isoxazoline and isoxazole scaffolds were previously studied in liquid crystal compounds [22]. The incidence of the SmA mesophase was intensified due to the segregation effect of perfluorinated and hydrogenated chains present in **17b**. For **17c** the absence of LC properties could be related to the disrupt the planarity and electronic conjugation between the phenyl rings caused by the inversion of isoxazoline ring – its isomer **17a**, despite is monotropic behavior displayed SmA mesophase.

A similar liquid crystal behavior can be seen in amides **19** and **21** where the isomers differ only in the relative orientation of the isoxazoline ring to the amide group, while the amide group in **19** can resonate to the nitrogen atom in the isoxazoline ring, **21** does not.

All analogous compounds such as thiourea **18a**,**b**, containing an isoxazole ring, displayed enantiotropic SmA and N mesophases, confirming that the isoxazole ring favors the formation of a stable mesophase. It is interesting to notice that the nematic mesophase appeared only in compounds with the isoxazole ring (**18a**, **18c** and **20**), confirming that a longitudinal diffusion is favored for this type of LC compounds [19].

For the amides an SmA mesophase was found in **19**, **20, 22** and **24**. The liquid crystal mesophase ranges for these amides are dependent on the 5-membered ring connecting the aryl groups. Thus, **19** and **20** display an SmA mesophase range (Δ*T*) of 6 °C and 69 °C, respectively. Another example are amides **21** and **22**. While **21** is not a LC, **22** displays a range of a mesophase of >42 °C. Examples above are representatives of the influence of 5-membered heterocycle on the mesomorphic behavior. Thus, isoxazoline derivatives induce the formation of an SmA mesophase by lateral diffusion, while isoxazole derivatives favor the formation of a nematic mesophase by longitudinal diffusion, along SmA mesophase [27].

In addition, for amide **19**, a second smectic mesophase was identified by POM as the monotropic SmC phase (Table 1, Figure 2). The transition to the SmC phase was not found in DSC analysis (Figure 3), due to its low transition enthalpy value. The transition from SmA to SmC, passing through broken fan-shaped focal conic texture for compound **19** is shown in Figure 2. Amide **20** displayed polymorphism Cr → SmA → N → I.

The DSC graphs for amides **19**, **20** and **24** showed a reproducible behavior in successive heating curves. This fact was not observed in the thiourea series **17a–c** and **18a–c**. In this series, a loss of signals corresponding to thermal transitions was observed in the third and fourth curves on the DSC graphs. This finding is detailed on the DSC graph for compounds **17a**, **19** and **20** (see Figure 3). The thermal changes in DSC may be interpreted by intermolecular hydrogen bonds, which can be established between thiourea molecules in their solid state or in the liquid crystal mesophases. Liquid crystals containing hydrogen bonds may exhibit a wide variety of phase polymorphism depending on the length of the chain, type of bonding and the functional groups involved [12,28–30]. Many liquid crystalline compounds have been developed exploring the ability of hydrogen bonding formation between dissimilar moieties, such as alkyloxybenzoic acids [31] and alkylbenzoic and dodecane dicarboxylic acids [32].

**Figure 4:**
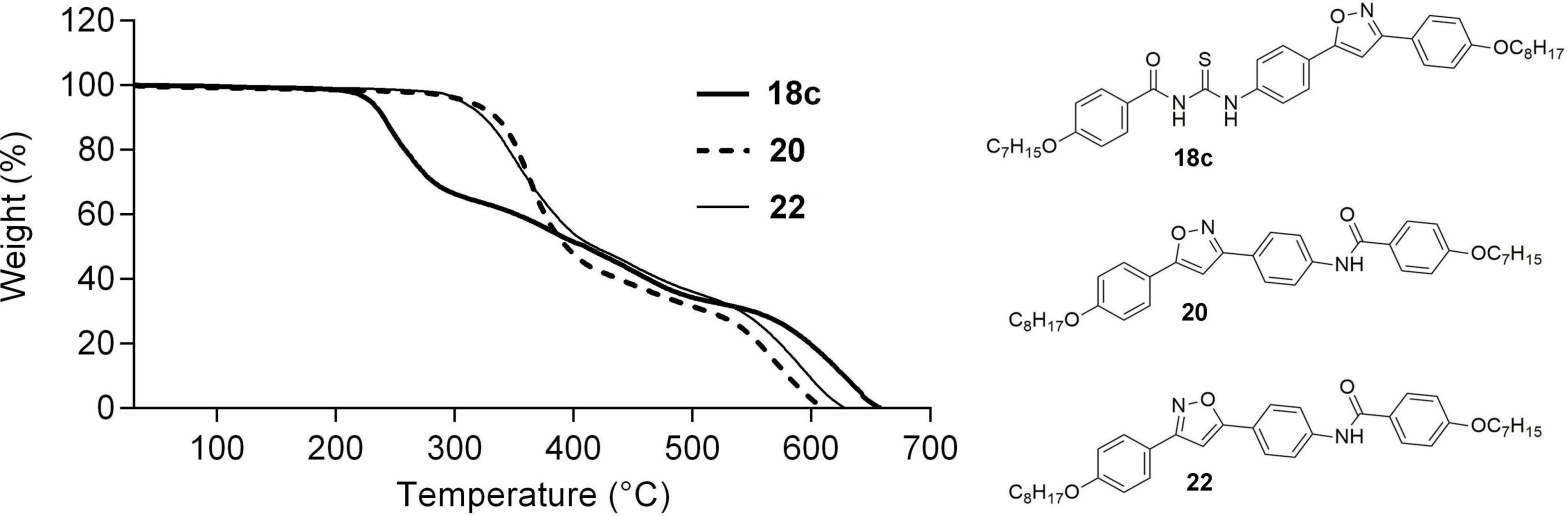
TGA analysis for thiourea **18c**; and amides **20** and **22**.

The thioureas, with a perfluorinated chain, **17b** and **18b,** presented thermal decomposition according to the DSC graphs, at temperatures above 200 °C. These compounds were not analyzed by termal gravimetric analysis (TGA) due to the possibility of hydrogen fluoride release in the system and the damage caused to the TGA. Relative to the isoxazole series, the thermal stability of two amides and one thiourea was assessed by TGA. Amides were found to be more stable than thioureas, where both amides, **20** and **22** only show thermal degradation above 300 °C, while thiourea **18c** decomposed above 200 °C (Figure 4). Also, amides **20** and **22**, which are isomers due to the change of the nitrogen and oxygen positioning in the isoxazole core, presented the same degradation behavior.

## Conclusion

The results reported here are the first examples for the synthesis and characterization of a novel series of thiourea and amide LCs with isoxazoline and isoxazole scaffolds. All reactions proceeded smoothly and with good yields. From TGA analysis thioureas are less stable than amides as evidenced by their degradation temperature. The SmA mesophase was predominant for both thioureas and amides and the nematic mesophase was observed only for thioureas and amides containing the isoxazole ring. The mesophase range was dependent on the nature of the 5-membered heterocycle. The isoxazole derivatives showed a larger mesophase range than the isoxazoline derivatives.

## Supporting Information

File 1 Experimental descriptions for the preparation of compounds and characterization data.

File 2 Differential scanning calorimetry plots of compounds.
