# Supplementary material for: The use of isoxazoline and isoxazole scaffolding in the design of novel thiourea and amide liquid-crystalline compounds
Source: Beilstein J Org Chem. 2020 Feb 6;16:175–84. doi: 10.3762/bjoc.16.20 (PMC7034240; doi:10.3762/bjoc.16.20)
Supplement: File 1 — Experimental descriptions for the preparation of compounds and characterization data. [file Beilstein_J_Org_Chem-16-175-s001.pdf]

BEILSTEIN JOURNAL OF ORGANIC CHEMISTRY

Supporting Information

for

The use of isoxazoline and isoxazole scaffolding in the design of novel thiourea and amide liquid-crystalline compounds

Itamar L. Gonçalves, Rafaela R. da Rosa, Vera L. Eifler-Lima and Aloir A. Merlo

Beilstein J. Org. Chem. **2020,** *16,* 175–184. doi:10.3762/bjoc.16.20

Experimental descriptions for the preparation of compounds and characterization data

License and Terms: This is a supporting information file under the terms of the Creative Commons Attribution License (https://creativecommons.org/licenses/by/4.0). Please note that the reuse, redistribution and reproduction in particular requires that the authors and source are credited.
The license is subject to the *Beilstein Journal of Organic Chemistry* terms and conditions: (https://www.beilstein-journals.org/bjoc)

Experimental section

General methods

The nuclear magnetic resonance spectra of hydrogen and carbon were recorded in a Bruker Ascend NMR with standard pulse sequences operating at 400 MHz for ^{1}H nuclei and 100 MHz for ^{13}C nuclei, using $CDCl_3$ as a solvent. Chemical shifts were reported as values (ppm) relative to TMS (0.0 ppm). The NMR multiplicities *brs*, *s*, *d*, *t*, *q*, and *m* represent broad singlet, singlet, doublet, triplet, quartet and multiplet, respectively. HRMS measurements were performed with a micro QTOF Q-III (Bruker Daltonics) mass spectrometer. The mass spectrometer was operated using ESI as ionization source. FT-IR spectra were obtained with a Shimadzu equipment operating in ATR mode. The melting points and mesophase transition temperatures and textures of the samples were measured on a Mettler Toledo FP82HT Hot Stage FP90 Central Processor and DSC 2910 TA Instruments.

General preparation of thioureas and amides

To a round bottom flask charged with 66 mg (0.28 mmol) of 4-heptyloxybenzoic acid was added 2 ml of thionyl chloride and this was refluxed at 85 ºC for 2 hours. After the excess of thionyl chloride was distilled off under reduced pressure, the residue comprising crude 4-heptyloxybenzoyl chloride was solubilized in 1 ml of acetone. Next, 0.30 mmol of ammonium thiocyanate (23 mg) or potassium cyanate (24 mg) was added, and the solution heated at 60 ºC for 40 minutes. Following this, the amines previously obtained [1,2] (0.28 mmol) and solubilized in acetone were added in a dropwise mode and heating was maintained for 60 minutes. The reaction solution was poured into water at room temperature and the precipitate was filtered and recrystallized in ethanol.

4-(*n*-Heptyloxy)-*N*-({4-[5-(4-octyloxyphenyl)-4,5-dihydroisoxazol-3-yl]phenyl}carbamothioyl)benzamide (**17a**): yield: 57%, melting point 157 ºC, recrystallized from ethanol. ^{1}H NMR (400 MHz, $CDCl_3$): 0.95 – 0.84 (m, 6H); 1.52 – 1.19 (m, 17H); 1.87 – 1.67 (m, 5H); 3.35 (dd, J = 16.6 Hz, J = 8.6 Hz, 1H); 3.72 (dd, 1H, J = 16.6 Hz, J = 10.9 Hz); 3.95 (t, J = 6.6 Hz, 2H); 4.03 (t, J = 6.5 Hz, 2H); 5.69 (dd, J = 10.7, 8.7 Hz, 1H); 6.89 (d, J = 8.7 Hz, 2H); 6.99 (d, J = 8.9 Hz, 2H); 7.30 (d, J = 8.7 Hz,

2H); 7.73 (d, J = 8.7 Hz, 2H); 7.88 – 7.80 (m, 4H); 9.06 (s, 1H); 12.87 (s, 1H). ^{13}C NMR (100 MHz, $CDCl_3$): 14.08; 14.11; 22.60; 22.66; 25.92; 26.03; 29.01; 29.03; 29.23; 29.35; 31.74; 31.81; 42.79; 68.10; 68.55; 82.77; 114.74; 114.97; 122.95; 123.82; 127.27; 127.38; 127.76; 129.73; 132.40; 139.16; 155.56; 159.25; 163.84; 166.45; 178.29. FT-IR (ATR) (cm^{-1}): 3147 (NH); 2914 (CH); 2848 (CH), 1666 (C=O); 1122 (C-O). HRMS/MS (m/z): calcd. $C_{38}H_{49}N_3O_4S$ [M+Cl]$^-$: 678.3127, found 678.3109.

4-(*n*-Heptyloxy)-*N*-({(4-[5-(4-(octyloxy)phenyl)isoxazol-3-yl]phenyl}carbamothioyl)benzamide (**18a**): yield: 61%, melting point: 134 ºC, recrystallized from ethanol. ^{1}H NMR (400 MHz, $CDCl_3$): 0.98 – 0.84 (m, 6H); 1.54 – 1.21 (m, 17H); 1.90 – 1.76 (m, 5H); 4.10 – 3.96 (m, 4H); 6.70 (s, 1H); 7.10 – 6.88 (m, 4H); 7.77 (d, J = 8.6 Hz, 2H); 7.87 (d, J = 8.8 Hz, 1H); 7.93 – 7.89 (m, 4H); 9.07 (s, 1H); 12.88 (s, 1H). ^{13}C NMR (100 MHz, $CDCl_3$): 14.09; 14.12; 22.61; 22.67; 25.93; 26.03; 29.02; 29.05; 29.18; 29.25; 29.36; 31.76; 31.82; 68.23; 68.55; 95.95; 114.93; 114.97; 119.98; 123.00; 124.00; 127.35; 127.44; 129.73; 139.11; 160.81; 162.18; 163.83; 166.45; 170.63; 178.30.FT-IR (ATR) (cm^{-1}): 3242 (NH); 2916 (CH); 2848 (CH); 1618 (C=O); 1247 (C-O). HRMS/MS (m/z): calcd. $C_{38}H_{47}N_3O_4S$ [M-H]$^-$: 640.3204, found 640.3217.

4-(*n*-Heptyloxy)-*N*-({(4-[5-(perfluorooctyl)-4,5-dihydroisoxazol-3-yl]phenyl}carbamothioyl)benzamide (**17b**): yield: 56%, melting point: 154 ºC, recrystallized from ethanol. ^{1}H NMR ($CDCl_3$, 400 MHz): 0.90 (t, *J* = 6.6 Hz, 1H); 1.41 – 1.27 (m, 7H); 1.52 – 1.42 (m, 3H); 1.87 – 1.77 (m, 2H); 3.67 (d, *J* = 9.1 Hz, 2H); 4.04 (t, *J* = 6.5 Hz, 2H); 5.33 – 5.15 (m, 1H); 7.00 (d, J = 8.8 Hz, 2H); 7.73 (d, J = 8.5 Hz, 2H); 7.91 – 7.82 (m, 4H); 9.05 (s, 1H); 12.92 (s, 1H). ^{13}C NMR (100 MHz, $CDCl_3$): 14.09; 22.62; 25.93; 29.03; 31.76; 35.94; 68.59; 115.02; 122.89; 123.89; 125.77; 127.61; 129.76; 140.00; 155.43; 163.93; 166.50; 178.39. FT-IR (ATR) (cm^{-1}): 3278 (NH); 2914 (CH); 2850 (CH); 1666 (C=O); 1195 (C-O); 1145 (C-O). HRMS/MS (m/z): calcd. $C_{32}H_{28}F_{17}N_3O_3S$ [M-H]$^-$: 856.1496, found 856.1495.

4-(*n*-Heptyloxy)-*N*-({(4-[5-(perfluorooctyl)isoxazol-3-yl]phenyl}carbamothioyl)benzamide (**18b**): yield: 60%, melting point 134 ºC, recrystallized from ethanol. ^{1}H NMR ($CDCl_3$, 400 MHz): 0.90 (t, *J* = 6.9 Hz, 3H); 1.42 – 1.28 (m, 6H); 1.52 – 1.42 (m, 2H); 1.88 – 1.78 (m, 2H); 4.05 (t, *J* = 6.5 Hz, 2H); 7.01 (d, *J* = 8.9 Hz, 2H); 7.08 (s, 1H); 7.91 – 7.84 (m, 4H); 7.95 (d, J = 8.7 Hz, 2H); 9.09 (s, 1H);

12.97 (s, 1H); ^{13}C NMR ($CDCl_3$, 100 MHz): 14.11; 22.62; 25.92; 29.03; 31.76; 68.56; 105.45; 114.99; 122.82; 124.19; 127.58; 129.76; 140.07; 158.95; 161.99; 163.89; 166.52; 178.42.FT-IR (ATR) (cm^{-1}): 3278 (NH); 2914 (CH); 2848 (CH); 1670 (C=O); 1199 (C-O); 1143 (C-O). HRMS/MS (m/z): calcd. $C_{32}H_{26}F_{17}N_3O_3S$ [M-H]$^-$: 854.1340, found 854.1352.

4-(*n*-Heptyloxy)-*N*-({(4-[3-(4-(octyloxy)phenyl)-4,5-dihydroisoxazol-5-yl]phenyl}carbamothioyl)benzamide (**17c**): yield: 58%, melting point 116 ºC, recrystallized from ethanol. ^{1}H NMR (400 MHz, $CDCl_3$): 1.00 – 0.83 (m, 6H); 1.54 – 1.22 (m, 18H); 1.87 – 1.74 (m, 4H); 3.32 (dd, *J* = 16.5, 8.1 Hz, 1H); 3.74 (dd, 1H, J = 16.6 Hz, J = 10.9 Hz); 3.95 (t, J = 6,6 Hz, 2H); 4.10 – 3.93 (m, 4H); 5.70 (dd, J = 10.7, 8.7 Hz, 1H); 6.91 (d, *J* = 8.8 Hz, 2H); 6.99 (d, *J* = 8.8 Hz, 2H); 7.44 (d, *J* = 8.5 Hz, 2H); 7.62 (d, *J* = 8.7 Hz, 2H); 7.74 (d, *J* = 8.4 Hz, 2H); 7.85 (d, *J* = 8.8 Hz, 2H); 9.02 (s, 1H); 12.71 (s, 1H). ^{13}C NMR (100 MHz, $CDCl_3$): 14.04; 14.05; 22.58; 22.64; 25.93; 26.02; 28.99; 29.06; 29.21; 29.34; 31.74; 31.80; 43.45; 68.22; 68.59; 81.79; 114.52; 114.78; 115.00; 120.43; 121.74; 123.19; 124.28; 126.45; 126.74; 128.30; 128.92; 129.70; 137.63; 139.64; 155.75; 160.83; 163.82; 166.41; 178.60.FT-IR (ATR) (cm^{-1}): 3223 (NH); 2916 (CH); 2848 (CH); 1641 (C=O); 1247 (C-O). HRMS/MS (m/z): calcd. $C_{38}H_{49}N_3O_4S$ [M-H]$^-$: 642.3360, found 642.3378.

4-(*n*-Heptyloxy)-*N*-({(4-[3-(4-(octyloxy)phenyl)isoxazol-5-yl]phenyl}carbamothioyl)benzamide (**18c**): yield: 62%, melting point: 141 ºC, recrystallized from ethanol. ^{1}H NMR (400 MHz, $CDCl_3$): 0.96 – 0.86 (m, 6H); 1.55 – 1.24 (m, 18H); 1.89 – 1.78 (m, 4H); 4.09 – 3.98 (m, 4H); 6.79 (s, 1H); 7.05 – 6.96 (m, 4H); 7.80 (d, J = 8.7 Hz, 2H); 7.90 – 7.84 (m, 4H); 7.94 (d, J = 8.7 Hz, 2H); 9.09 (s, 1H); 12.95 (s, 1H). ^{13}C NMR (100 MHz, $CDCl_3$): 14.09; 14.12; 22.60; 22.67; 29.02; 29.21; 29.25; 29.37; 31.75; 31.82; 68.14; 68.60; 97.39; 114.83; 114.96; 121.24; 122.88; 123.91; 125.54; 126.37; 128.17; 129.73; 139.22; 160.65; 162.68; 163.84; 166.45; 169.29; 178.21. FT-IR (ATR) (cm^{-1}): 3402 (NH); 2918 (CH); 2848 (CH); 1668 (C=O); 1251 (C-O). HRMS/MS (m/z): calcd. $C_{38}H_{47}N_3O_4S$ [M-H]$^-$: 640.3204, found 640.3227.

4-*n*-Heptyloxy-*N*-{4-[5-(4-octyloxyphenyl)-4,5-dihydroisoxazol-3-yl]phenyl}benzamide (**19**): yield: 42%, melting point: 201 ºC, recrystallized from ethanol. ^{1}H NMR (400 MHz, $CDCl_3$): 0.94 – 0.84 (m, 6H); 1.51 – 1.19 (m, 18H); 1.87 – 1.71 (m, 4H); 3.32 (dd, J = 16.6, 8.7 Hz, 1H); 3.71 (dd, *J* = 16.6, 10.8 Hz, 1H); 3.95 (t, J = 6.6 Hz, 2H); 4.02 (t, J =

6.6 Hz, 2H); 5.67 (dd, J = 10.7, 8.7 Hz, 1H); 6.89 (d, J = 8.6 Hz, 2H); 6.96 (d, J = 8.7 Hz, 2H); 7.30 (d, J = 8.7 Hz, 2H); 7.72 – 7.65 (m, 4H); 7.84 – 7.81 (m, 2H). ^{13}C NMR (100 MHz, $CDCl_3$): 14.07; 22.61; 22.66; 25.99; 26.07; 29.04; 29.17; 29.24; 29.29; 29.37; 31.78; 31.83; 42.96; 68.21; 68.40; 82.59; 114.64; 114.83; 119.91; 125.48; 126.59; 127.39; 127.68; 128.95; 132.61; 139.86; 155.77; 159.31; 162.42; 165.21. FT-IR (ATR) (cm^{-1}): 3317 (NH); 2914 (CH); 2848 (CH); 1645 (C=O); 1251 (C-O). HRMS/MS (m/z): calcd. $C_{37}H_{48}N_2O_4$ $[M-H]^-$: 583.3530, found 583.3557.

4-*n*-Heptyloxy-*N*-{4-[5-(4-octyloxyphenyl)isoxazol-3-yl]phenyl}benzamide (**20**): yield: 53%, melting point: 202 ºC, recrystallized from ethanol. ^{1}H NMR (400 MHz, $CDCl_3$): 0.93 – 0.86 (m, 6H); 1.58 – 1.20 (m, 18H); 1.86 – 1.74 (m, 4H); 4.05 – 3.99 (m, 4H); 6.68 (s, 1H); 7.01 – 6.95 (m, 4H); 7.78 – 7.73 (m, 4H); 7.89 – 7.80 (m, 4H). FT-IR (ATR) (cm^{-1}): 3363 (NH); 2910 (CH); 2845 (CH); 1654 (C=O); 1246 (C-O). HRMS/MS (m/z): calcd. $C_{37}H_{46}N_2O_4$ $[M-H]^-$: 581.3374, found 581.3374.

4-*n*-Heptyloxy-*N*-{4-[3-(4-octyloxyphenyl)-4,5-dihydroisoxazol-5-yl]phenyl}benzamide (**21**): yield: 58%, melting point: 197 ºC, recrystallized from ethanol. ^{1}H NMR (400 MHz, $CDCl_3$): 0.99 – 0.79 (m, 6H); 1.60 – 1.19 (m, 18H); 1.89 – 1.72 (m, 4H); 3.30 (dd, J = 16.6, 8.7 Hz, 1H); 3.73 (dd, *J* = 16.6, 10.8 Hz, 1H); 3.08 – 3.93 (m, 4H); 5.68 (t, J = 9.5 Hz, 1H); 6.91 (d, J = 8.3 Hz, 2H); 6.95 (d, J = 8.4 Hz, 2H); 7.38 (d, J = 8.0 Hz, 2H); 7.67 – 7.56 (m, 4H); 7.74 (bs, 1H); 7.82 (d, J = 8.2 Hz, 2H). ^{13}C NMR (100 MHz, $CDCl_3$): 14.06; 14.07; 22.61; 22.66; 25.99; 26.04; 29.04; 29.18; 29.23; 29.36; 31.78; 31.83; 43.43; 68.25; 68.38; 82.04; 114.60; 114.80; 120.41; 121.90; 126.79; 128.31; 128.90; 137.01; 138.06; 155.83; 150.83; 162.30; 165.23. FT-IR (ATR) (cm^{-1}): 3324 (NH); 2918 (CH); 2850 (CH); 1645 (C=O); 1249 (C-O). HRMS/MS (m/z): calcd. $C_{37}H_{48}N_2O_4$ $[M-H]^-$: 583.3530, found 583,3548.

4-*n*-Heptyloxy-*N*-{4-[3-(4-octyloxyphenyl)isoxazol-5-yl]phenyl}benzamide (**22**): yielding: 53%, melting point: 208 ºC, recrystallized from ethanol. ^{1}H NMR (400 MHz, $CDCl_3$): 1.05 – 0.82 (m, 6H); 1.67 – 1.23 (m, 18H); 1.95 – 1.73 (m, 1H); 4.14 – 3.97 (m, 4H); 6.76 (s, 1H); 7.06 – 6.95 (m, 4H); 7.94 – 7.74 (m, 8H). FT-IR (ATR) (cm^{-1}): 3358 (NH); 2912 (CH); 2843 (CH); 1656 (C=O); 1244 (C-O). HRMS/MS (m/z): calcd. $C_{37}H_{46}N_2O_4$ $[M-H]^-$: 581.3374, found 581.3378.

4-*n*-Nonyloxy-*N*-{4-[5-(4-octyloxyphenyl)-4,5-dihydroisoxazol-3-yl]phenyl}benzamide (**24**): yield: 71%, melting point: 192 ºC, recrystallized from ethanol. ^{1}H NMR (400 MHz, $CDCl_3$): 0,96 – 0,85 (m, 6H); 1,54 – 1,23 (m, 20H); 1,90 – 1,73 (m, 2H); 3,34 (dd, J = 16,6, 8,7 Hz, 1H); 3,74 (dd, *J* = 16,6, 10,8 Hz, 1H); 3,98 (t, *J* = 6,6 Hz, 2H); 4,05 (t, *J* = 6,6 Hz, 2H); 5,67 (dd, J = 10,7, 8,7 Hz, 1H); 6,92 (d, J = 8 Hz, 2H); 6,96 (d, J = 7,9 Hz, 2H); 7,33 (d, *J* = 9,4, 2H); 7,77 – 7,68 (m, 4H); 7,92 – 7,79 (m, 3H). FT-IR (ATR) (cm^{-1}): 3321 (NH); 2916 (CH); 2846 (CH); 1651 (C=O), 1251 (C-O). HRMS/MS (m/z): calcd. $C_{39}H_{52}N_2O_4$ $[M+Cl]^-$: 647.3610, found 647.3634.

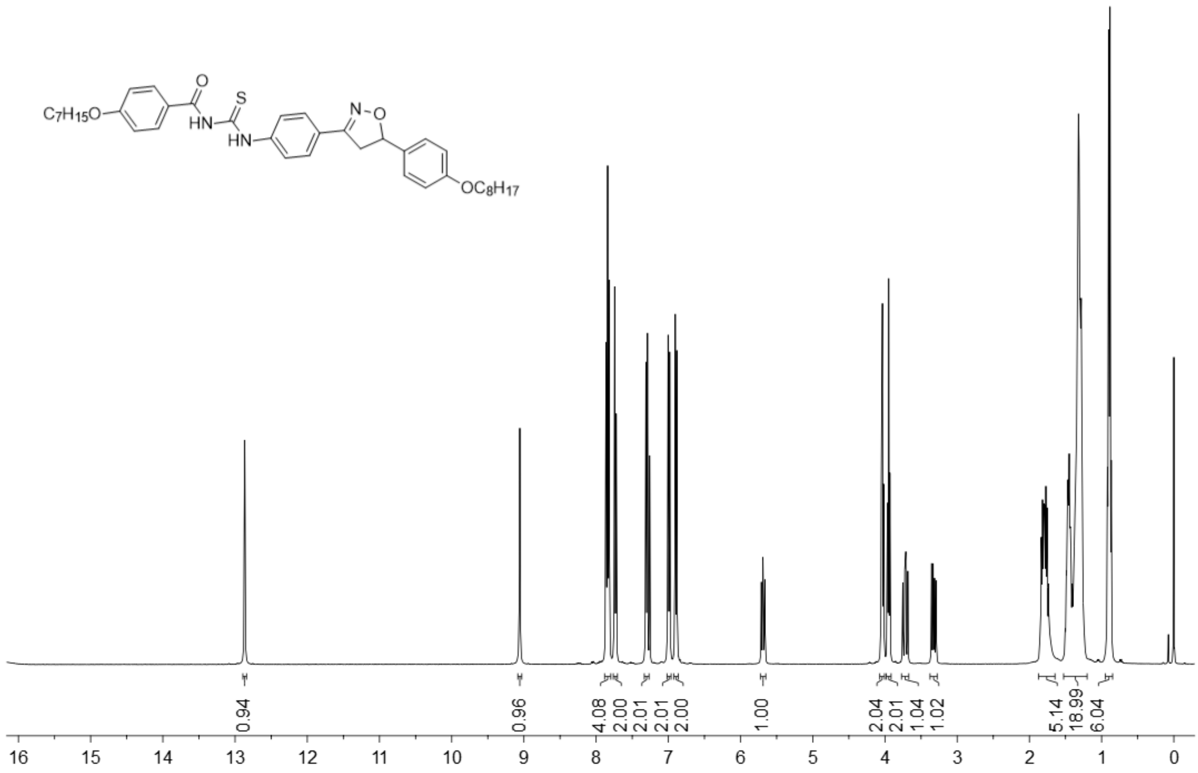

Figure S1 – ^{1}H NMR spectrum of compound **17a** ($CDCl_3$, 400 MHz)

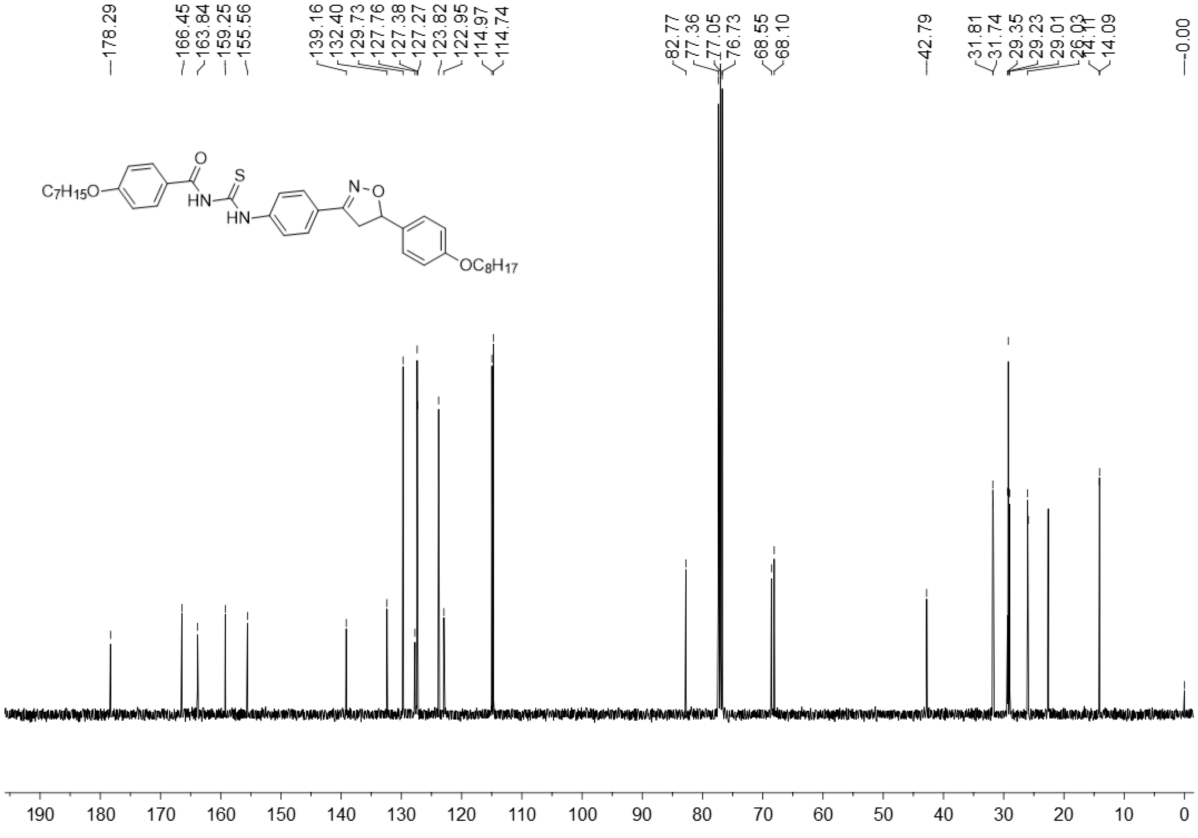

Figure S2 – ^{13}C NMR spectrum of compound **17a** ($CDCl_3$, 100 MHz)

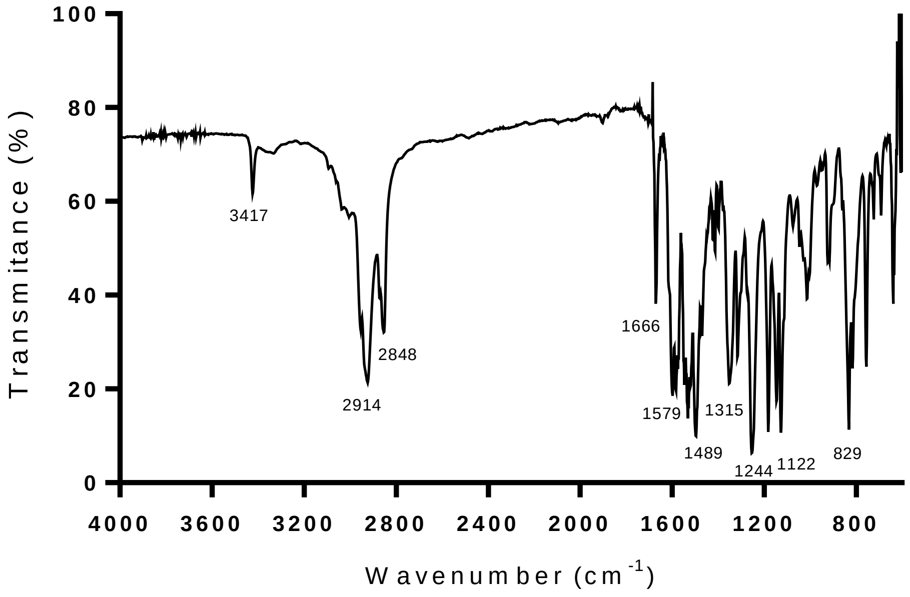

Figure S3 –FT IR spectrum of compound **17a**

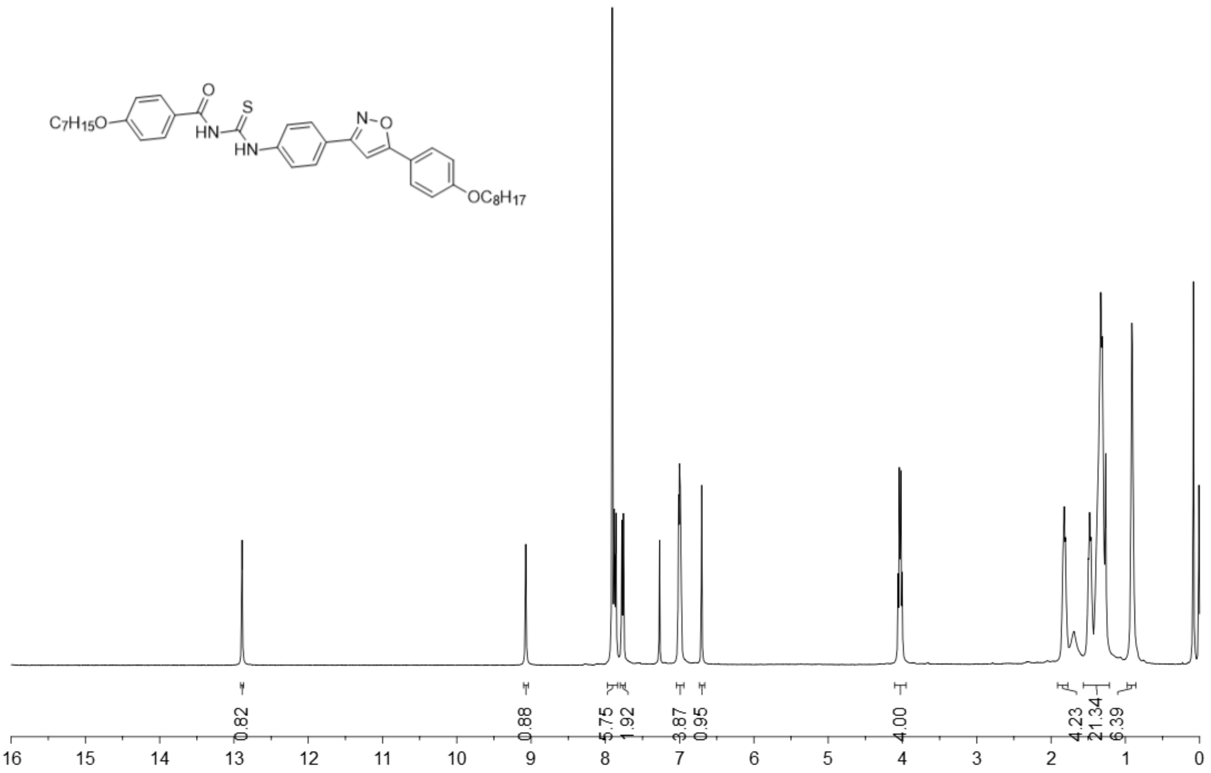

Figure S4 – ^{1}H NMR spectrum of compound **18a** ($CDCl_3$, 400 MHz)

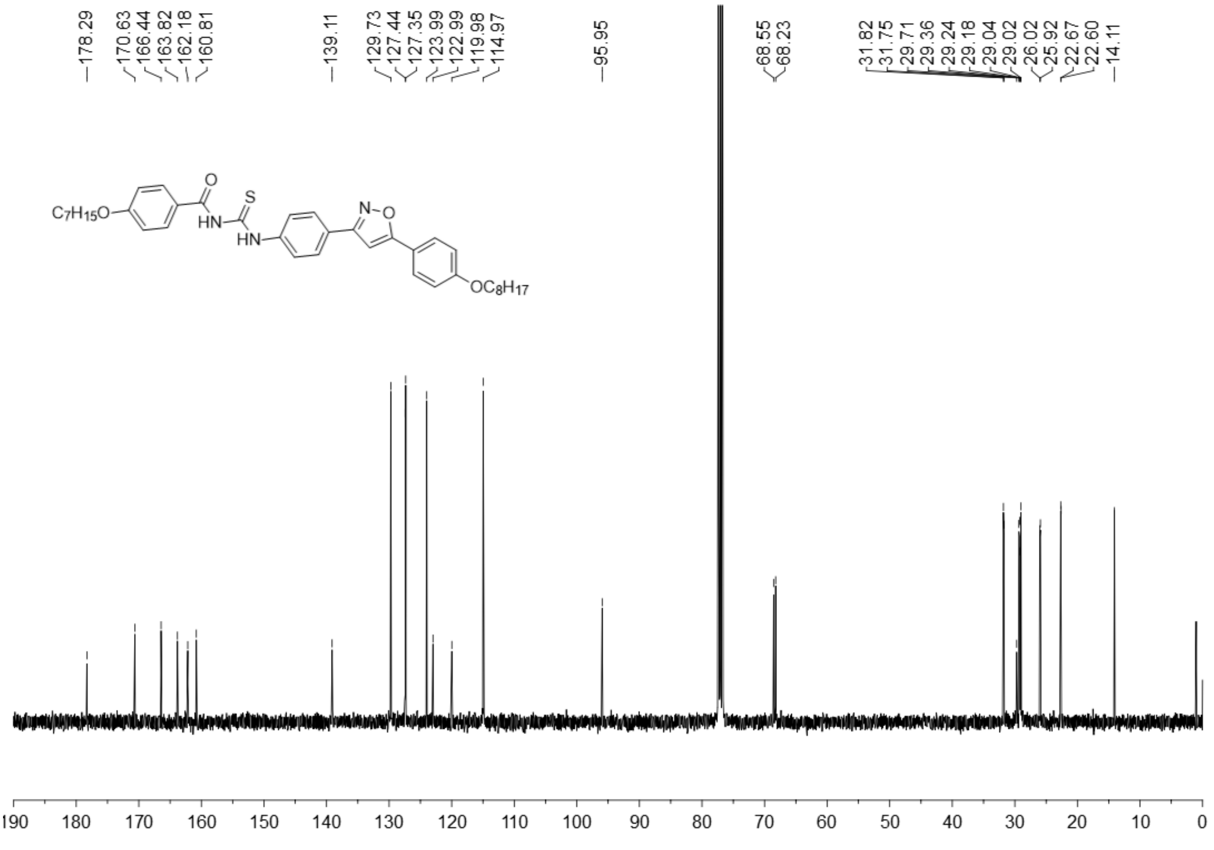

Figure S5 – ^{13}C NMR spectrum of compound **18a** ($CDCl_3$, 100 MHz)

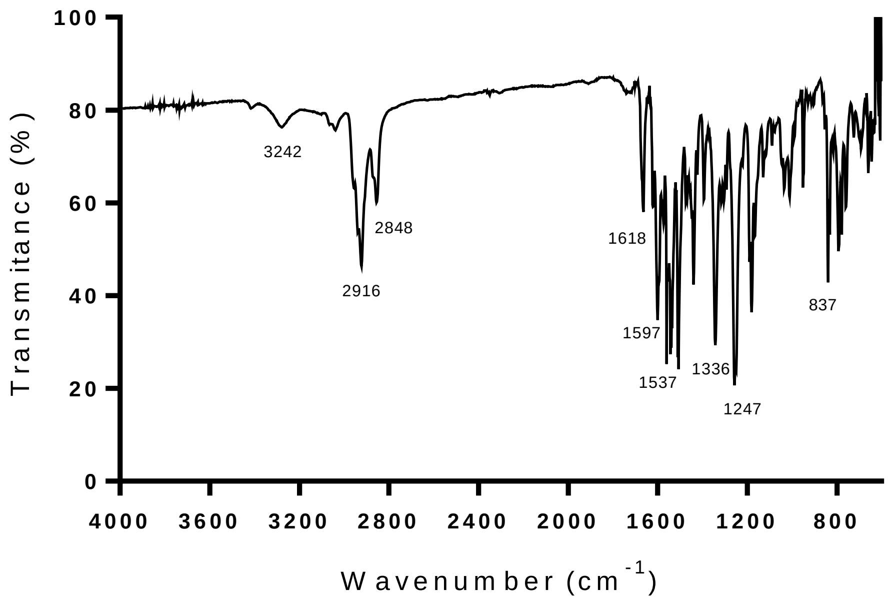

Figure S6 –FT IR spectrum of compound **18a**

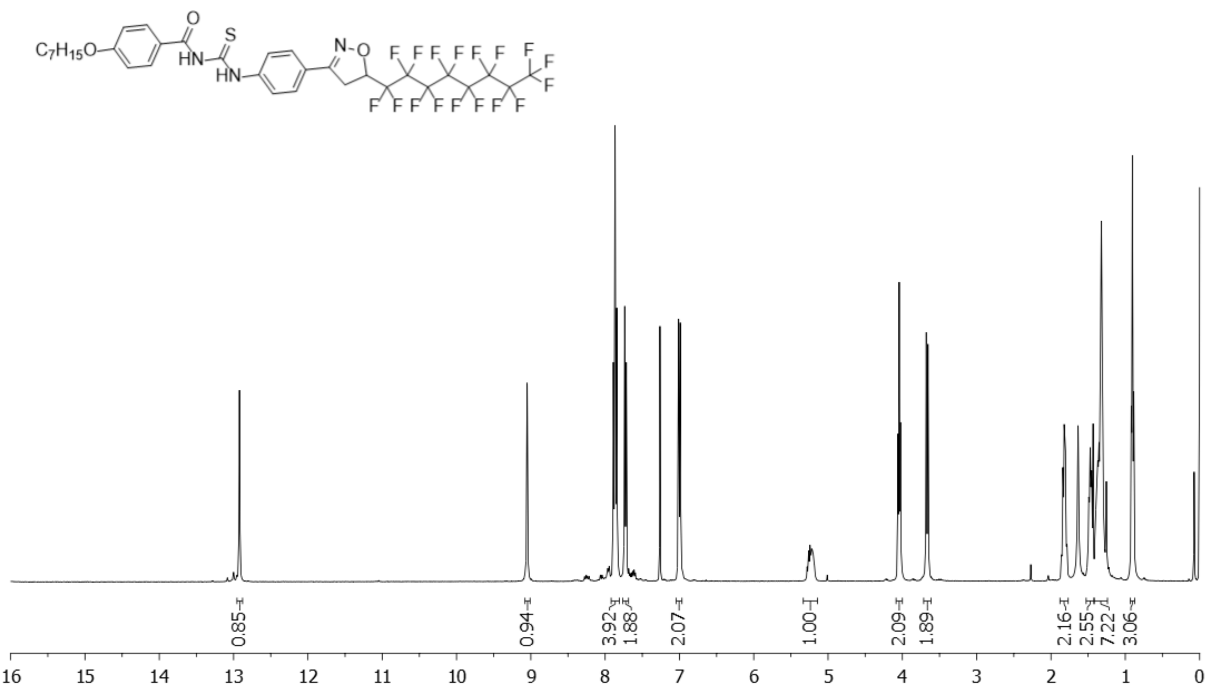

Figure S7 – ^{1}H NMR spectrum of compound **17b** ($CDCl_3$, 400 MHz)

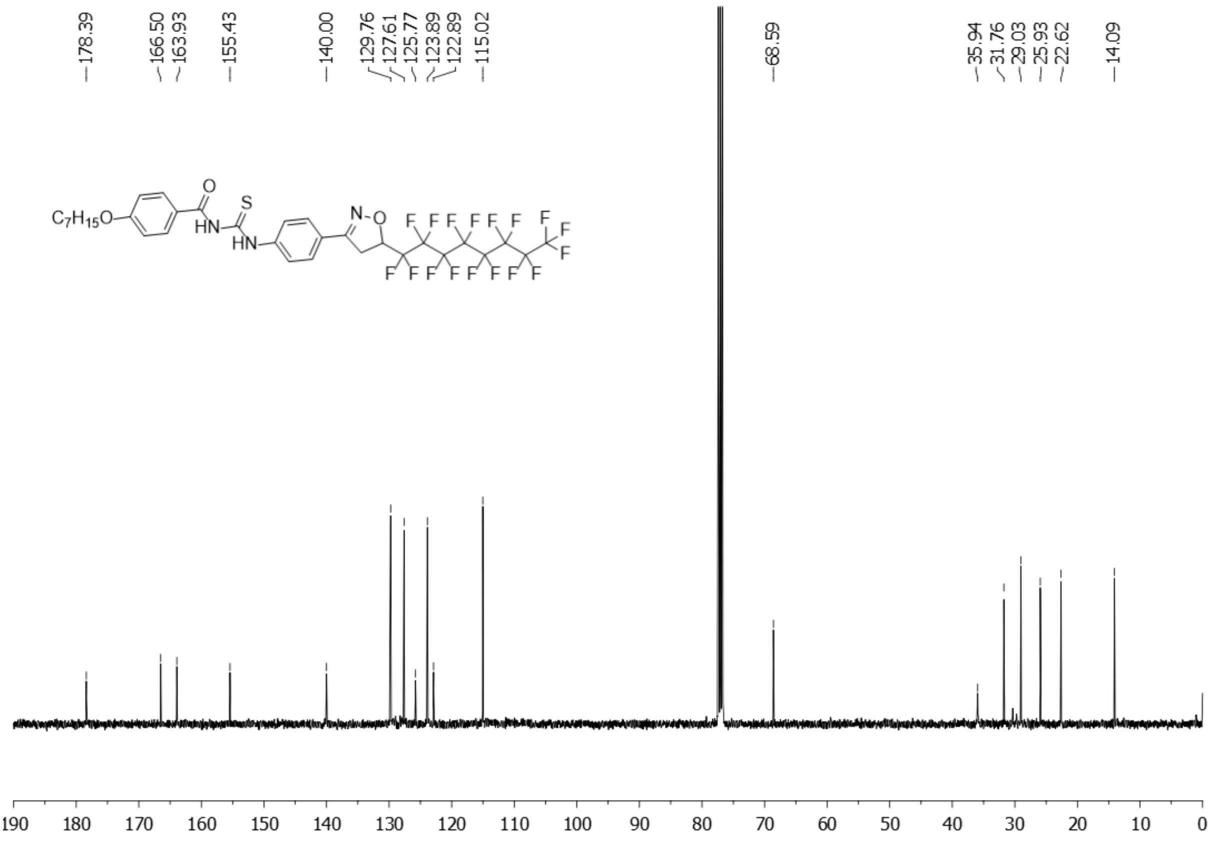

Figure S8 – ^{13}C NMR spectrum of compound **17b** ($CDCl_3$, 100 MHz)

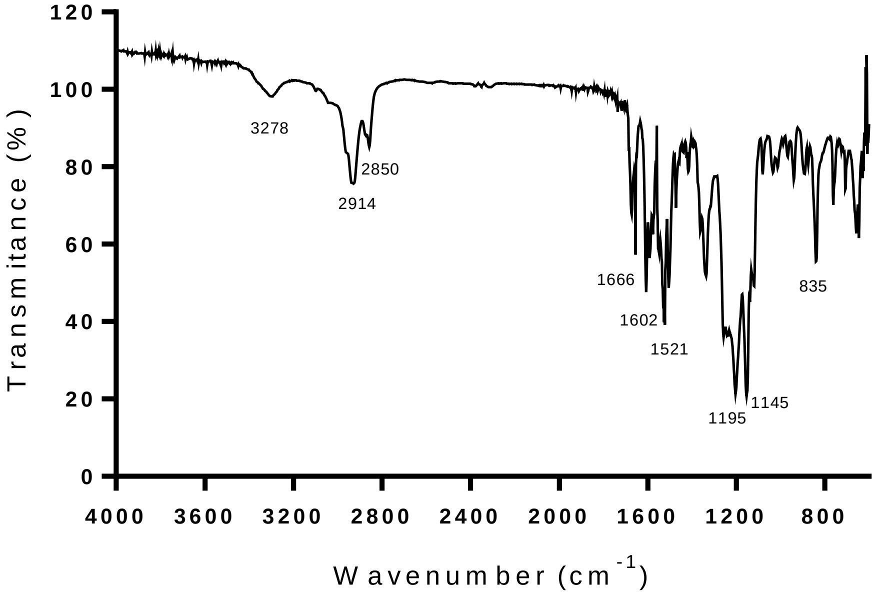

Figure S9 –FT IR spectrum of compound **17b**

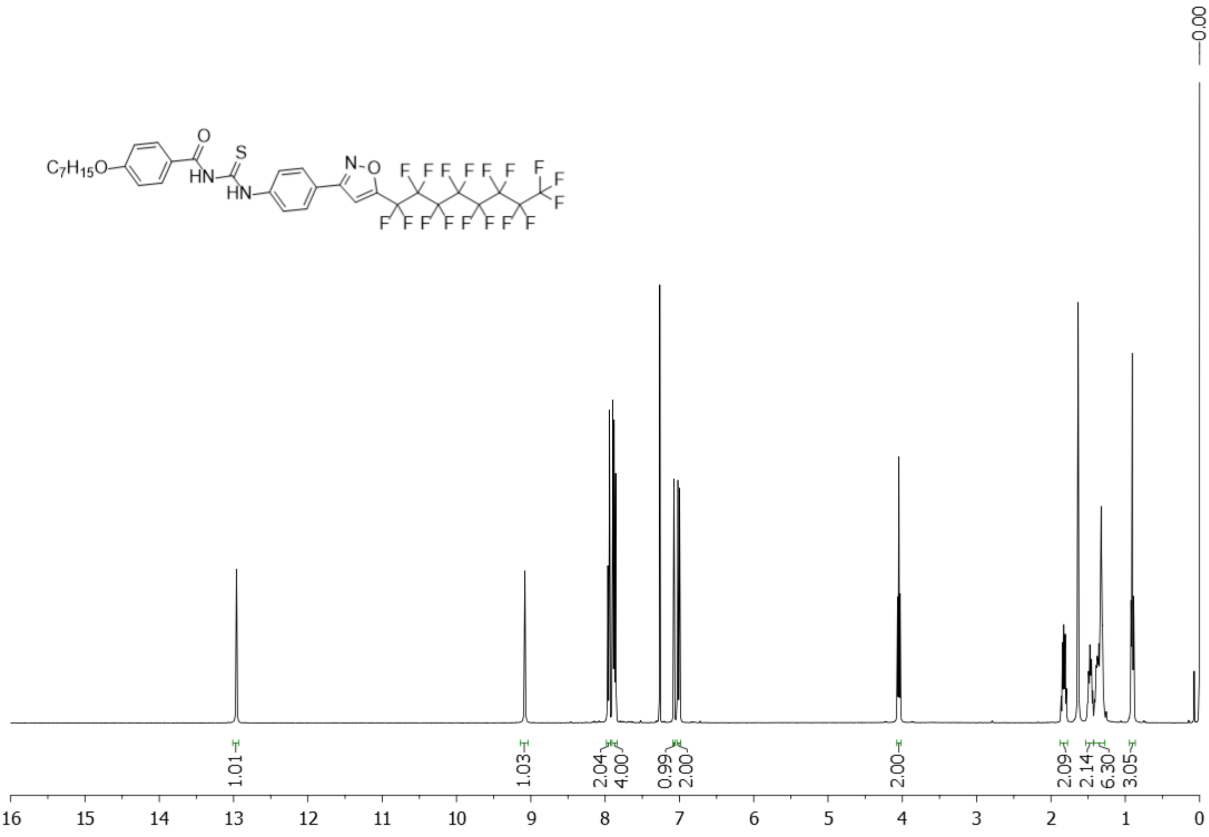

Figure S10 – ^{1}H NMR spectrum of compound **18b** ($CDCl_3$, 400 MHz)

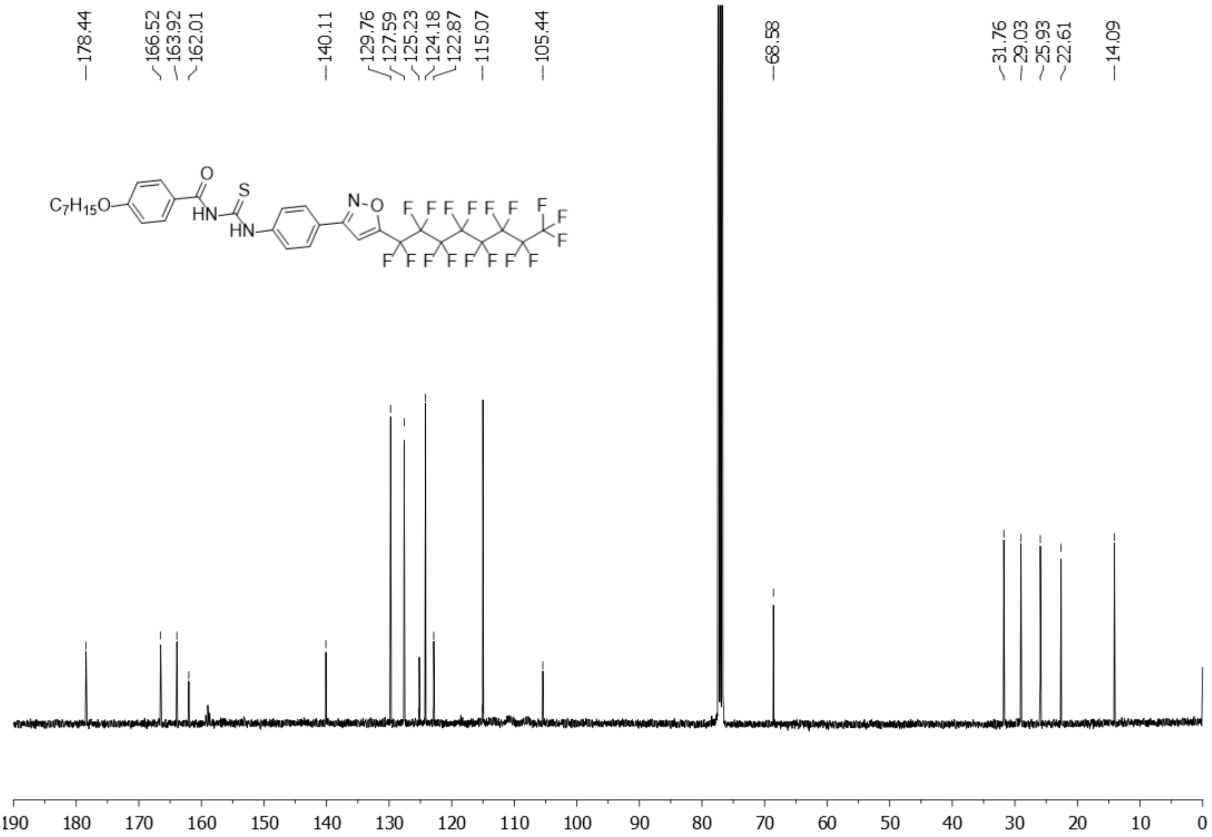

Figure S11 – ^{13}C NMR spectrum of compound **18b** ($CDCl_3$, 100 MHz)

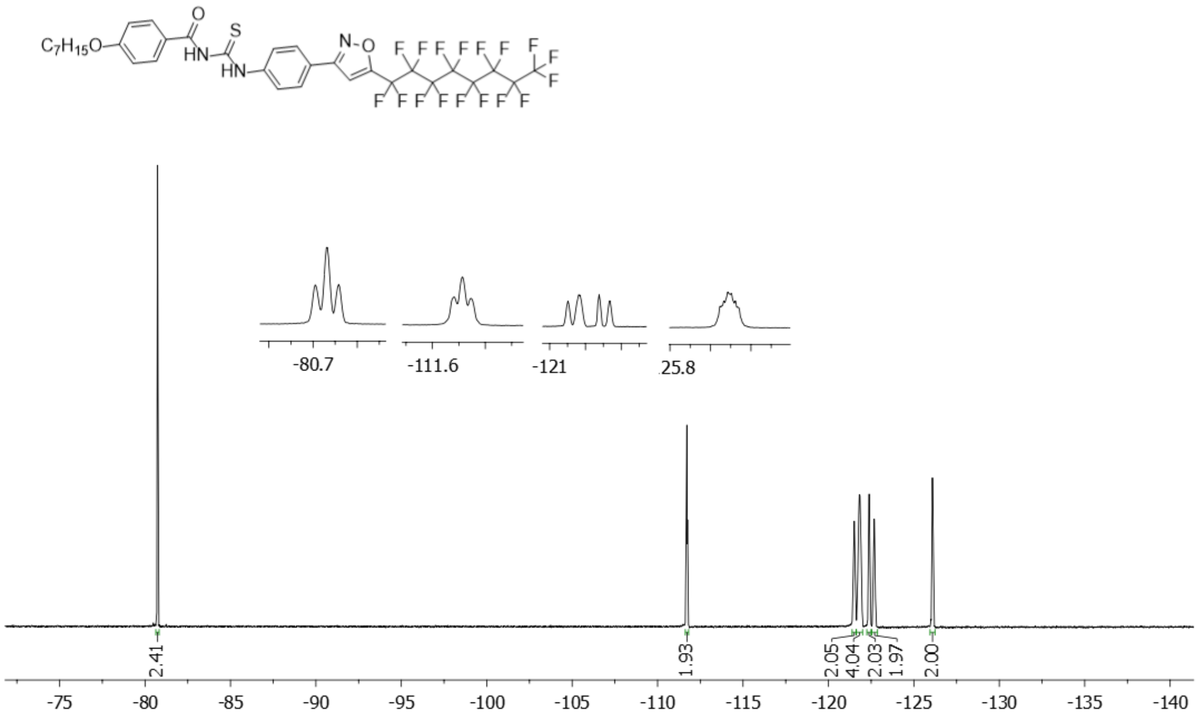

Figure S12 – ^{19}F NMR spectrum of compound **18b** ($CDCl_3$, 376 MHz)

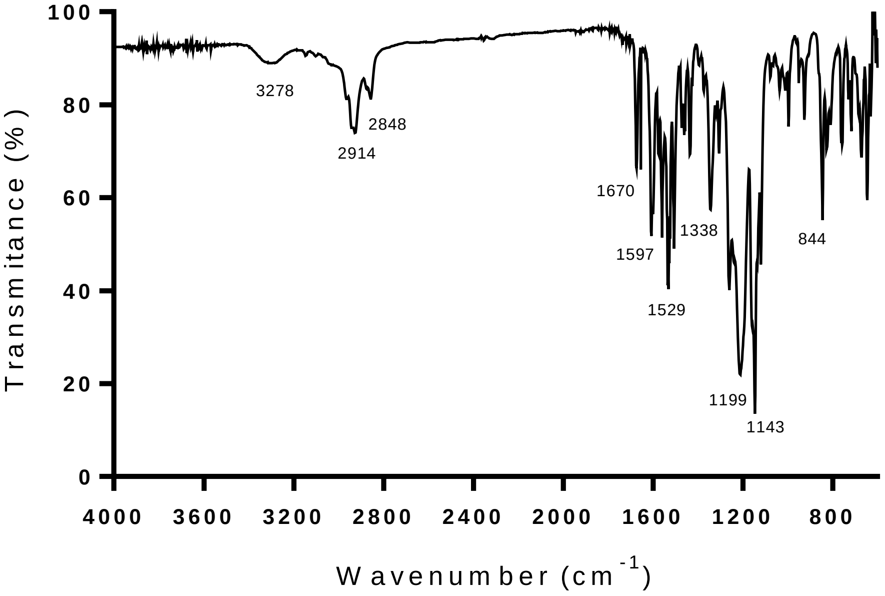

Figure S13 –FT IR spectrum of compound **18b**

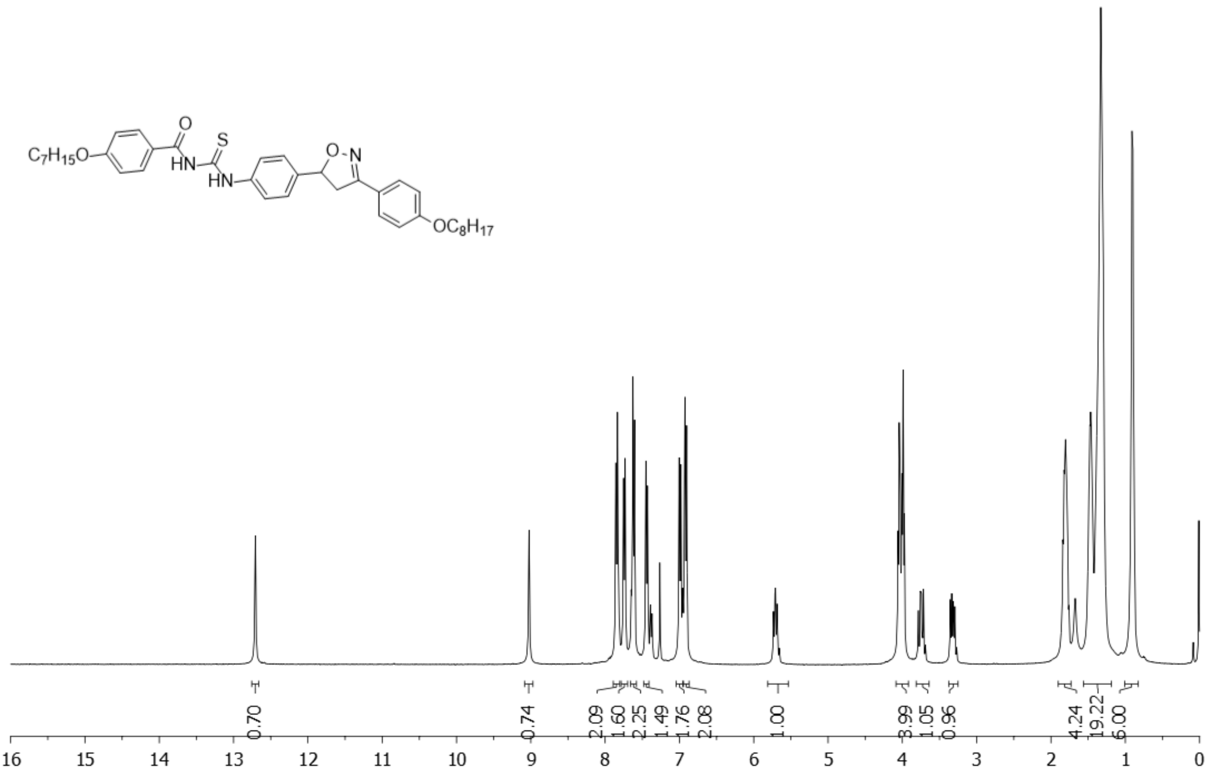

Figure S14 – ^{1}H NMR spectrum of compound **17c** ($CDCl_3$, 400 MHz)

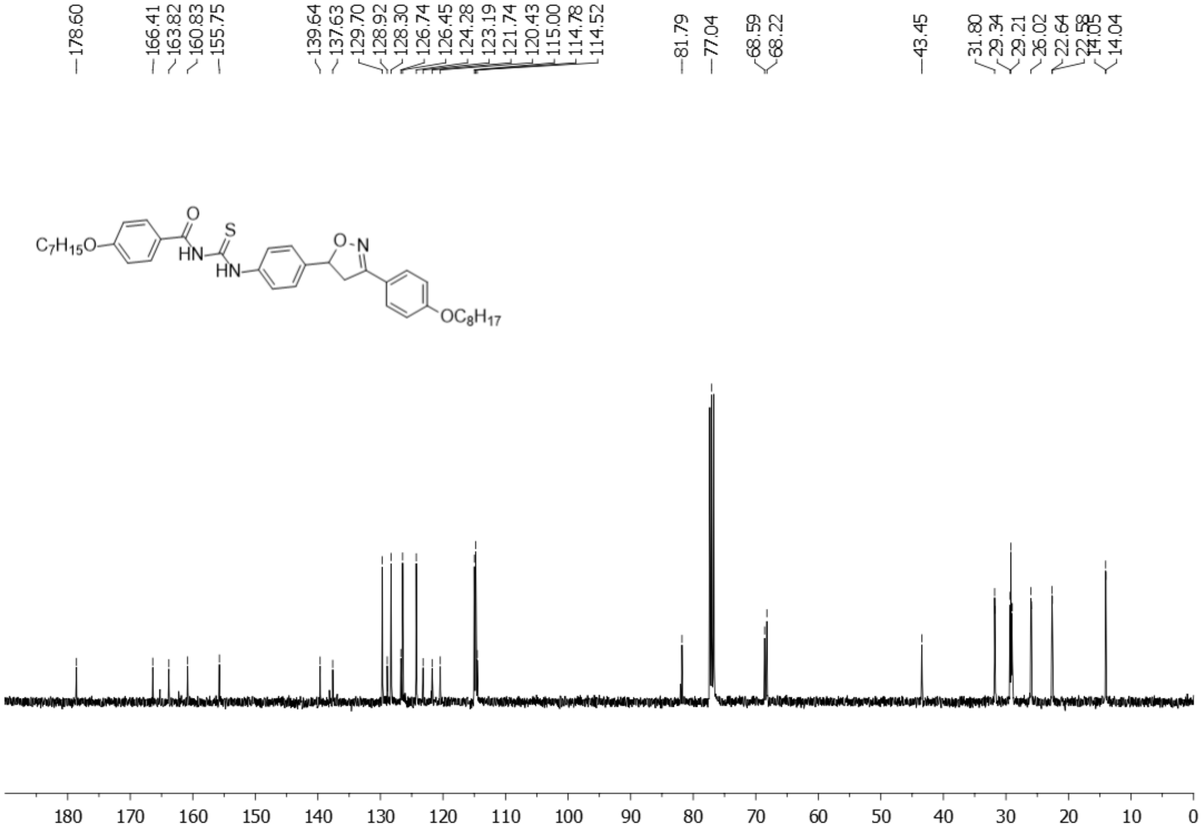

Figure S15 – ^{13}C NMR spectrum of compound **17c** ($CDCl_3$, 100 MHz)

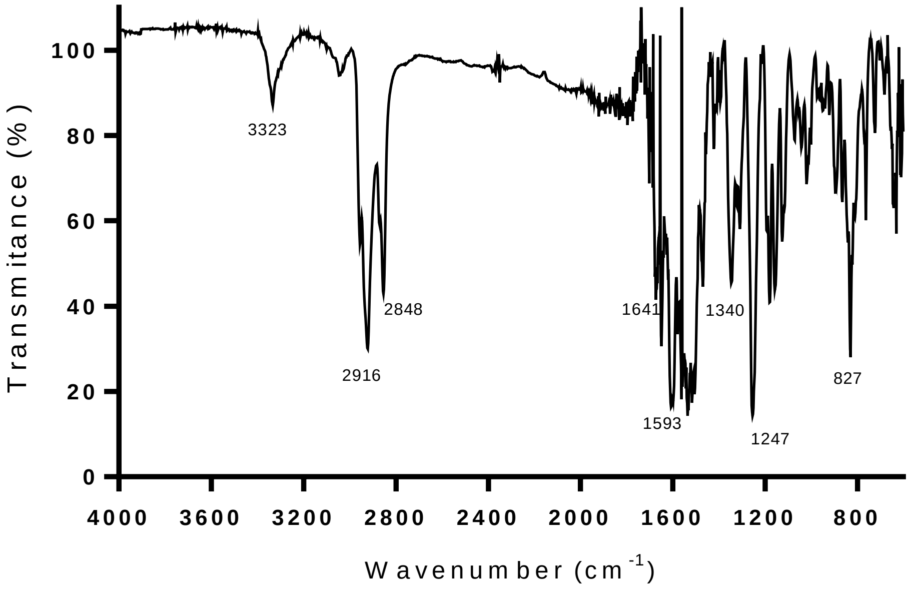

Figure S16 –FT IR spectrum of compound **17c**

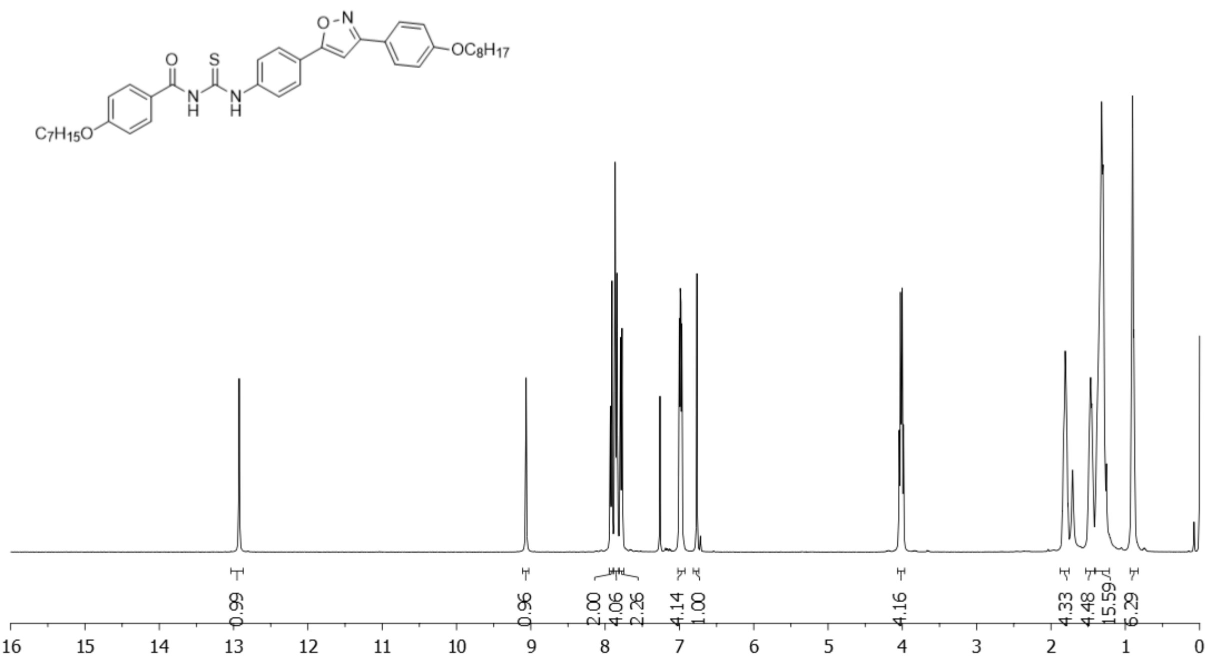

Figure S17 – ^{1}H NMR spectrum of compound **18c** ($CDCl_3$, 400 MHz)

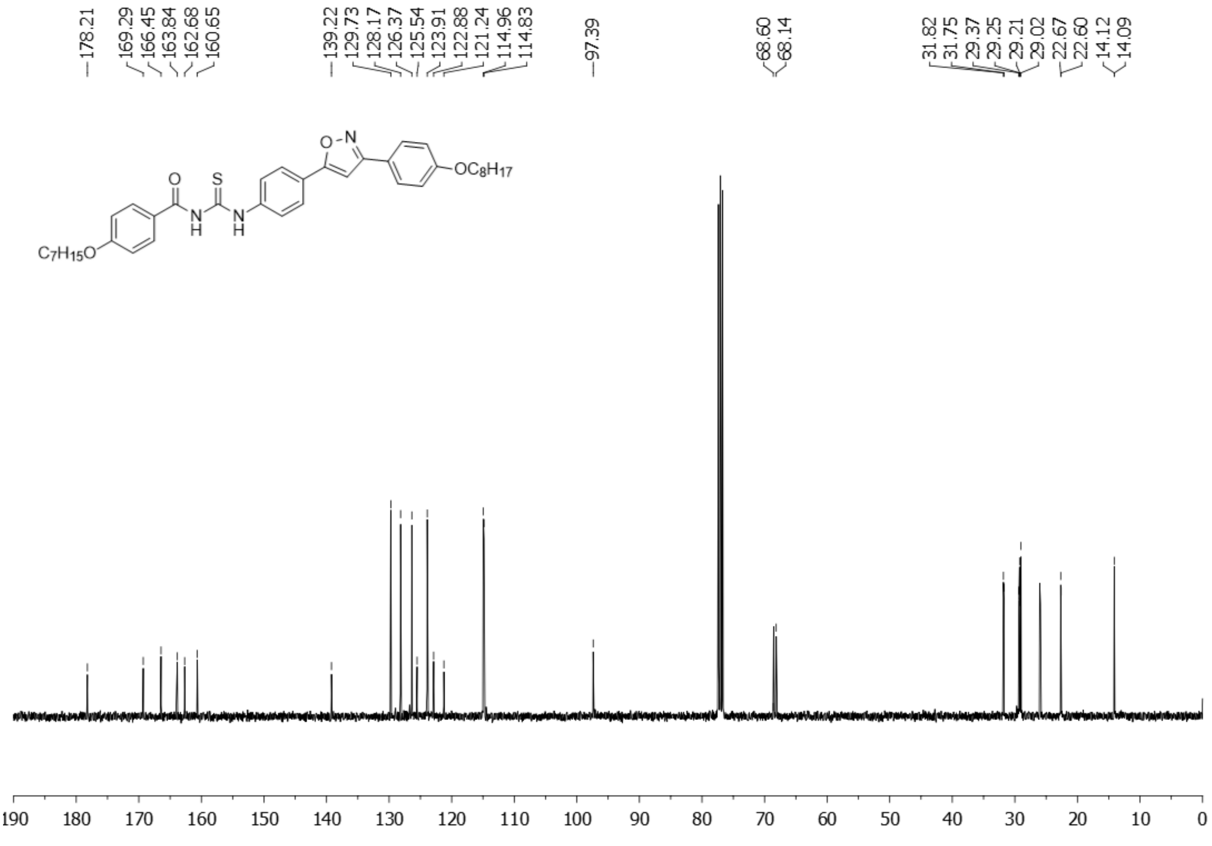

Figure S18 – ^{13}C NMR spectrum of compound **18c** ($CDCl_3$, 100 MHz)

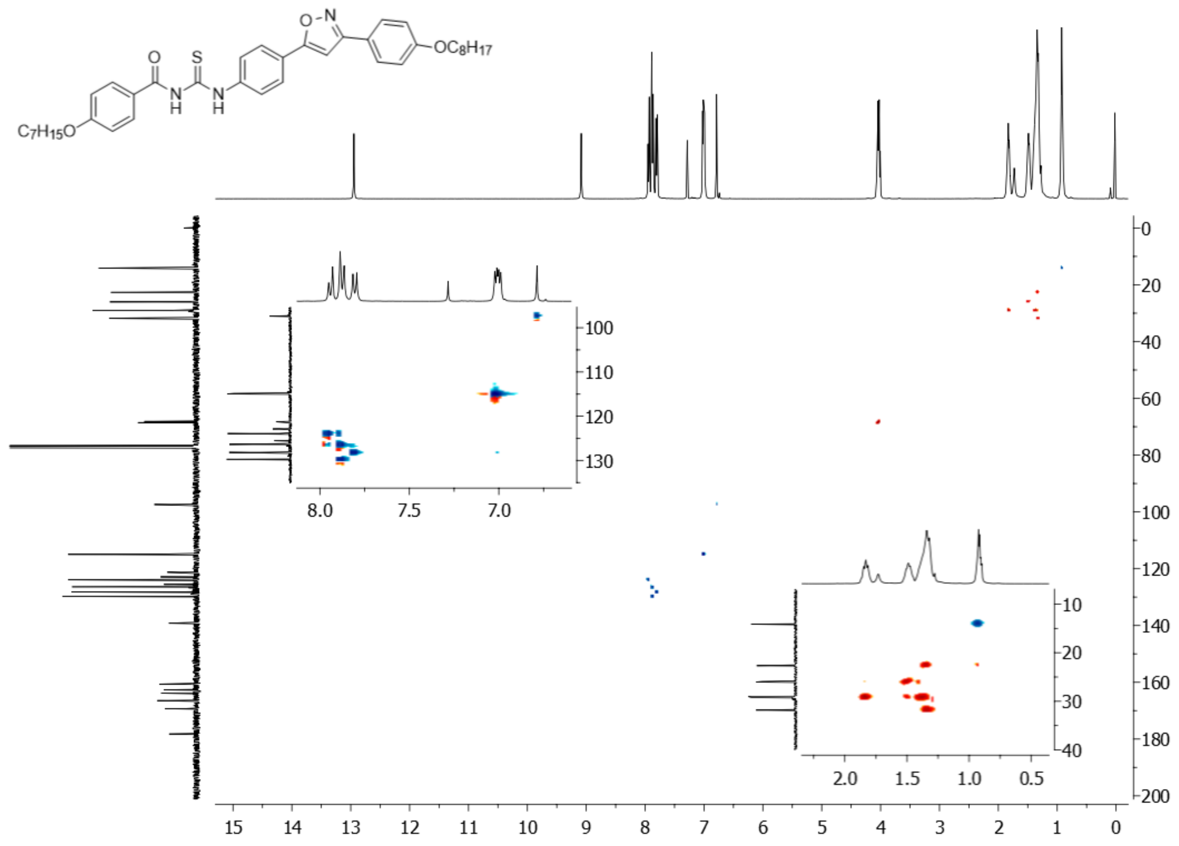

Figure S19 – HSQC experiment for compound **18c**

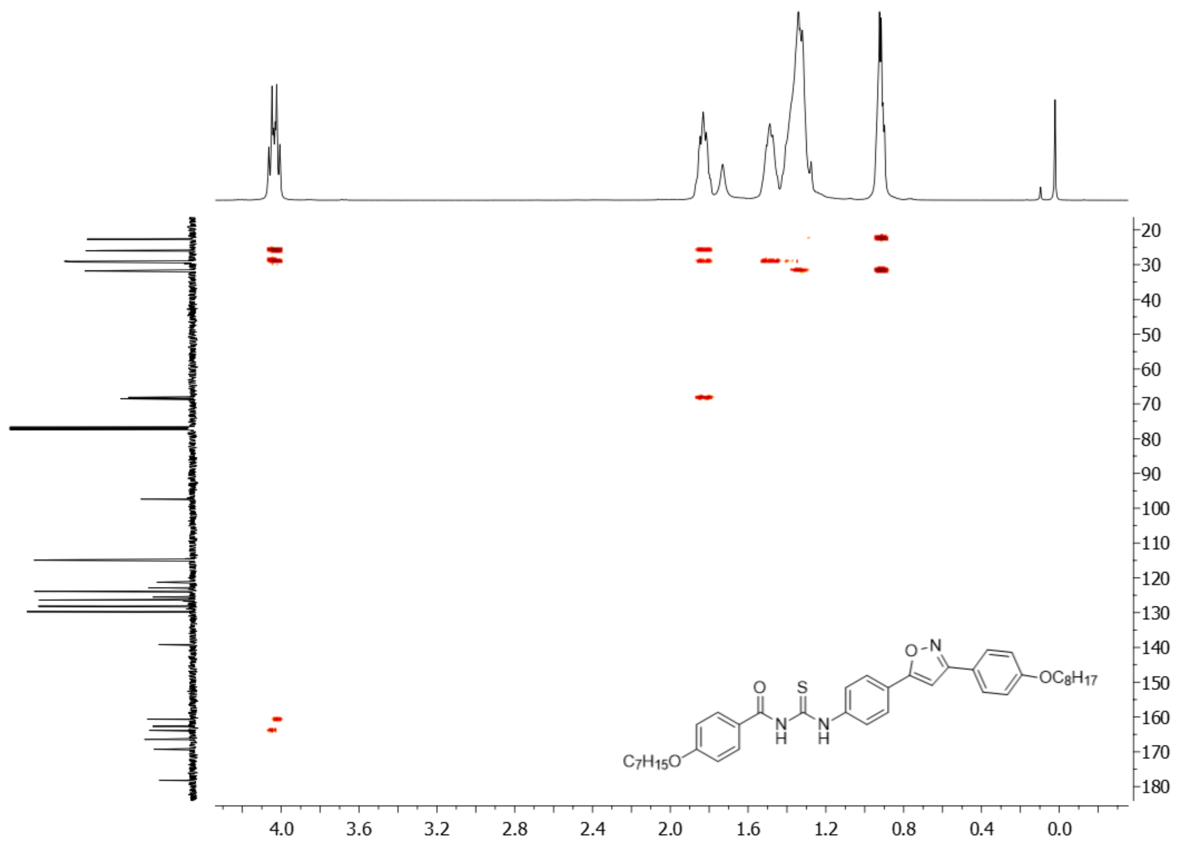

Figure S20 – Expansion aliphatic region of HMBC experiment for compound **18c**.

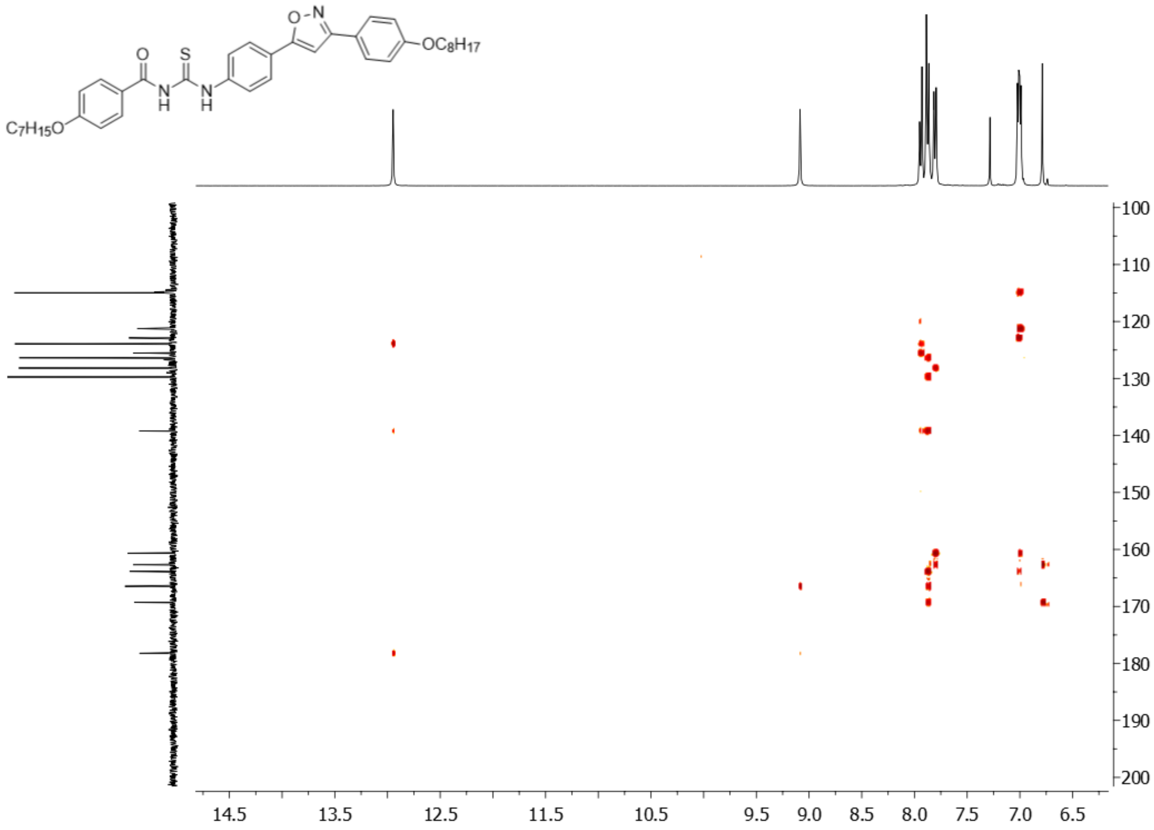

Figure S21 – Expansion aromatic region of HMBC experiment for compound **18c**.

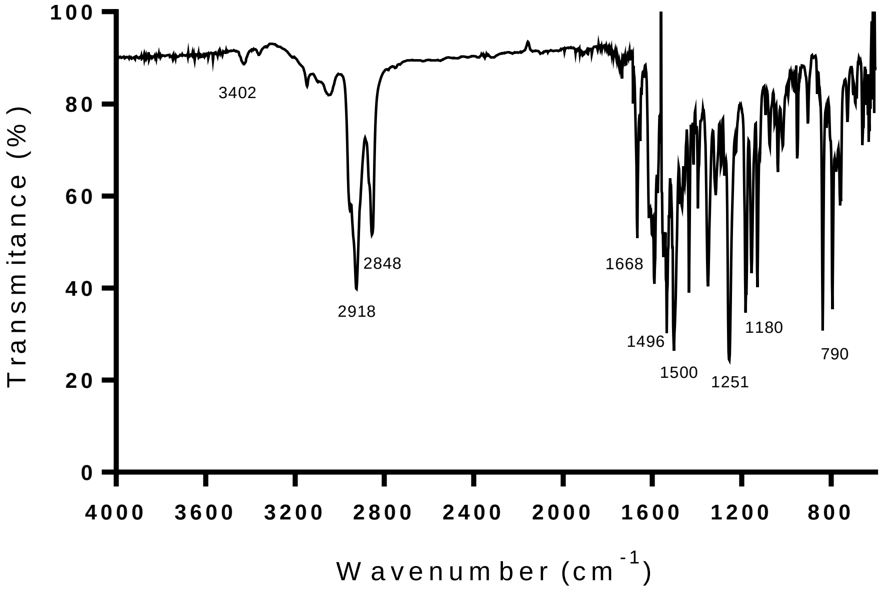

Figure S22 –FT IR spectrum of compound **18c**

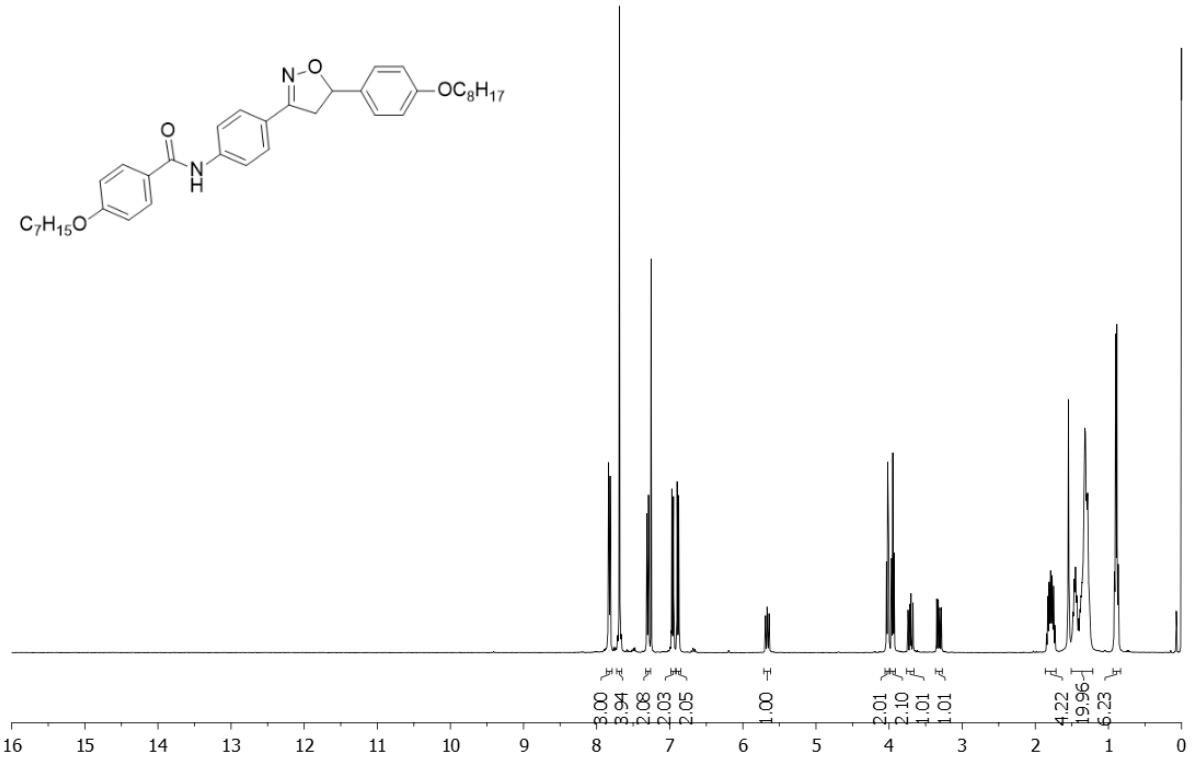

Figure S23 – ^{1}H NMR spectrum of compound **19** ($CDCl_3$, 400 MHz)

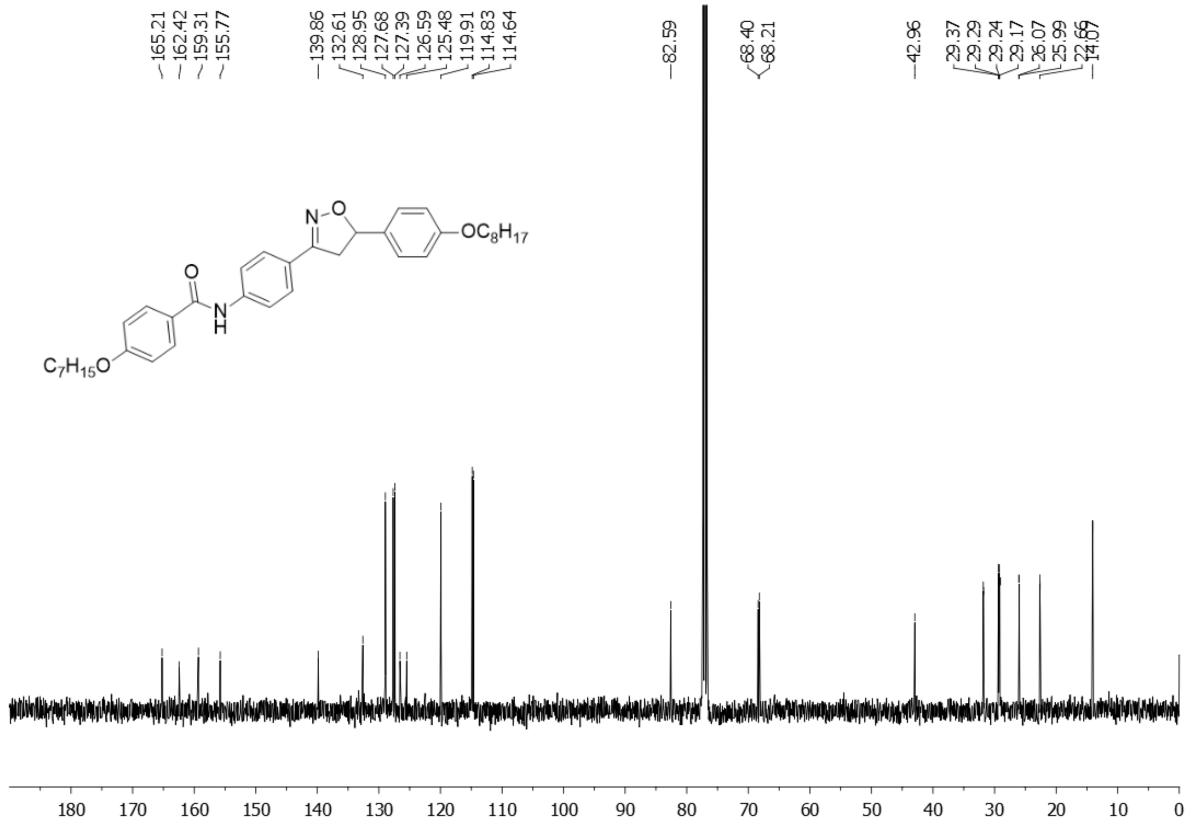

Figure S24 – ^{13}C NMR spectrum of compound **19** ($CDCl_3$, 100 MHz)

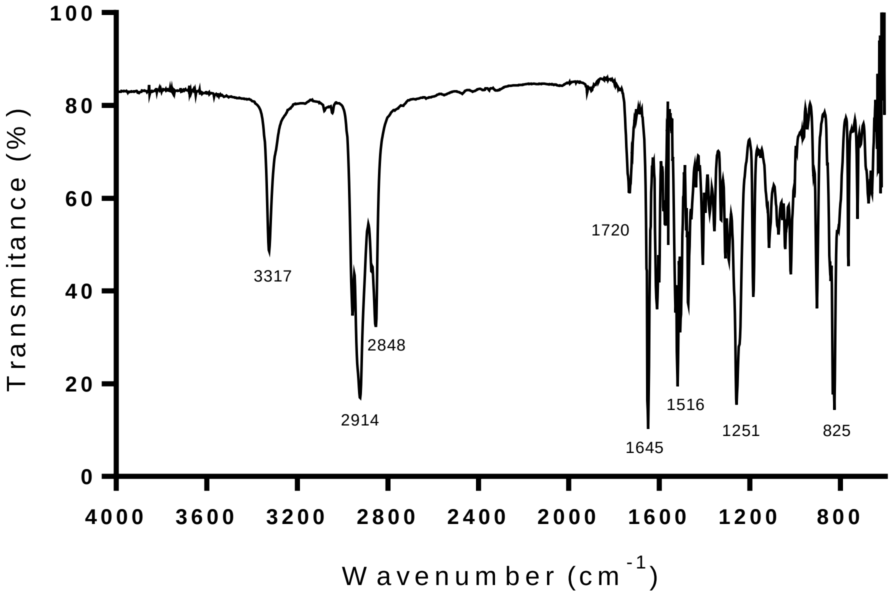

Figure S25 –FT IR spectrum of compound **19**

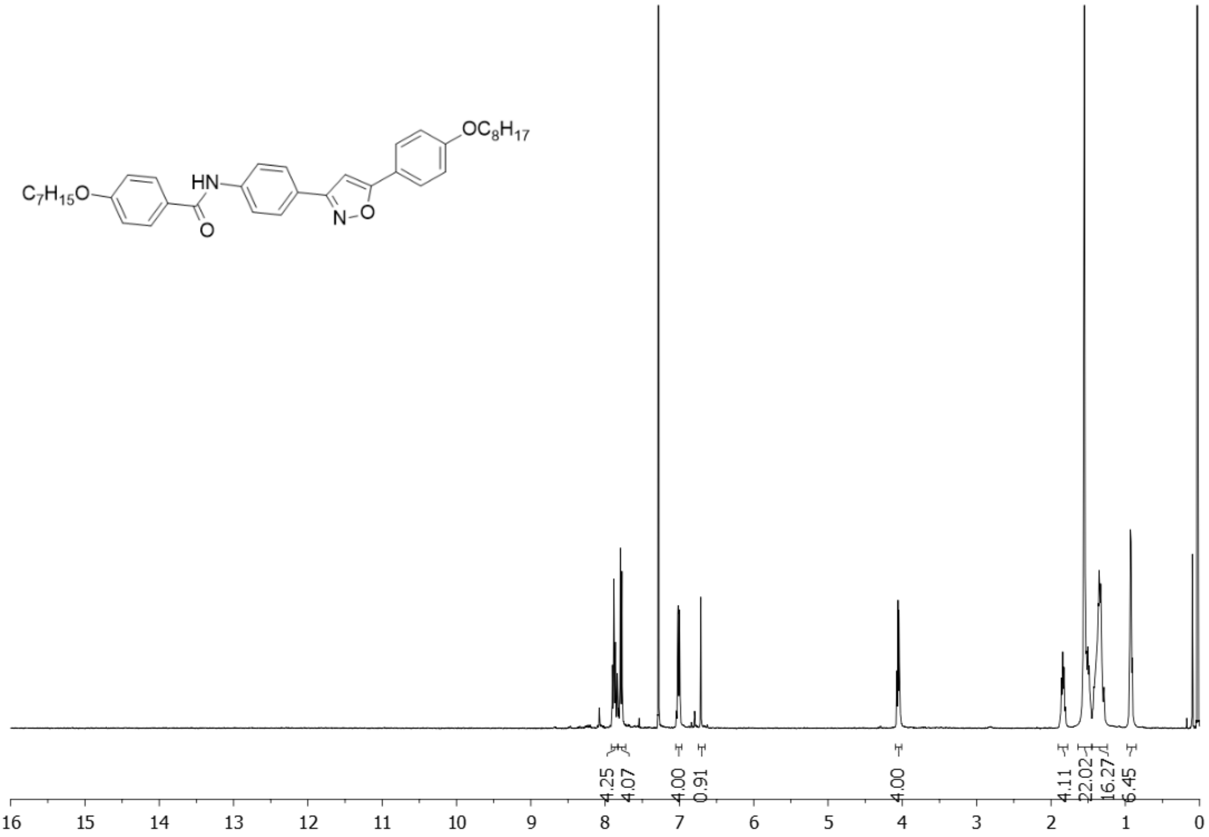

Figure S26 – ^{1}H NMR spectrum of compound **20** ($CDCl_3$, 400 MHz)

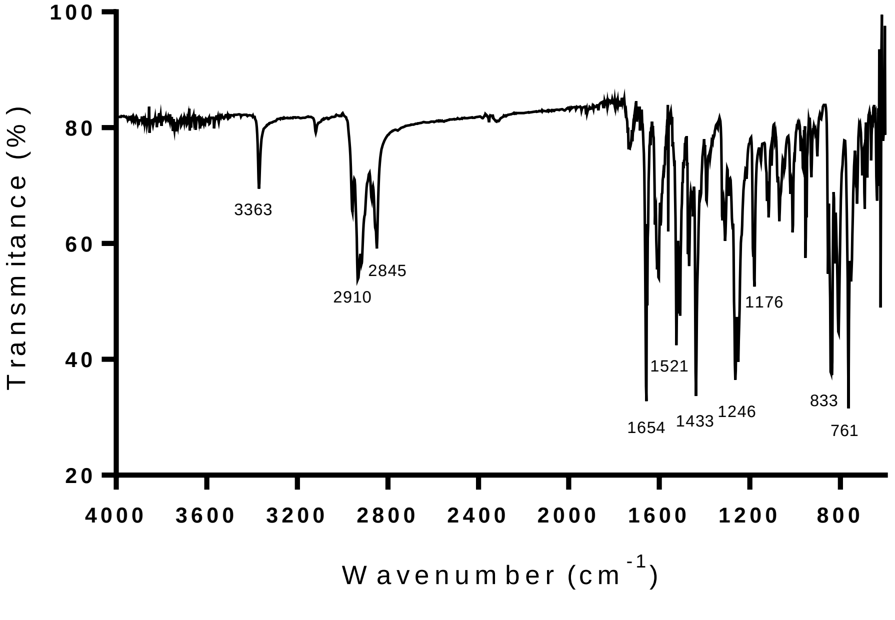

Figure S27 –FT IR spectrum of compound **20**

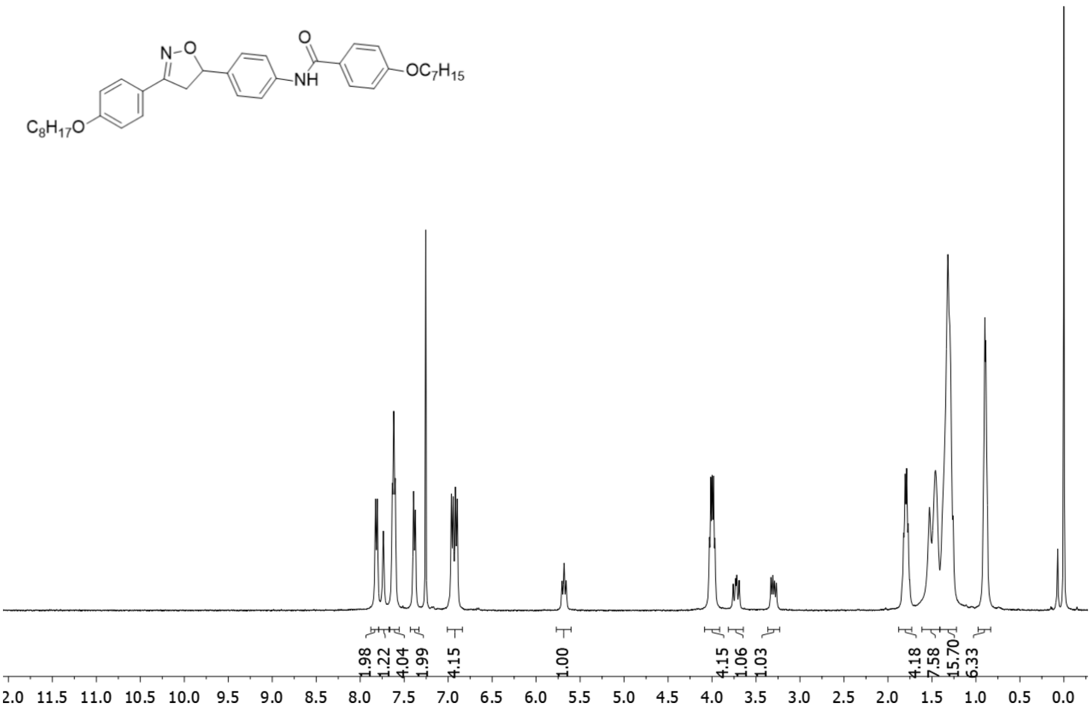

Figure S28 – ^{1}H NMR spectrum of compound **21** ($CDCl_3$, 400 MHz)

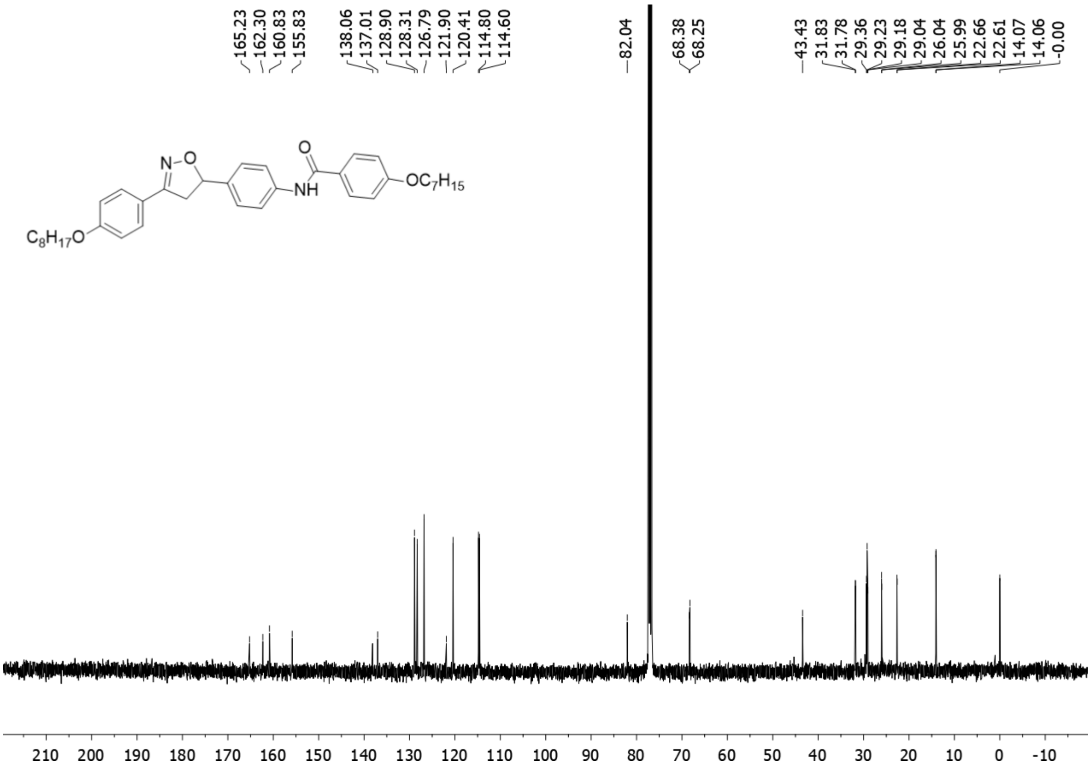

Figure S29 – ^{13}C NMR spectrum of compound **21** ($CDCl_3$, 100 MHz)

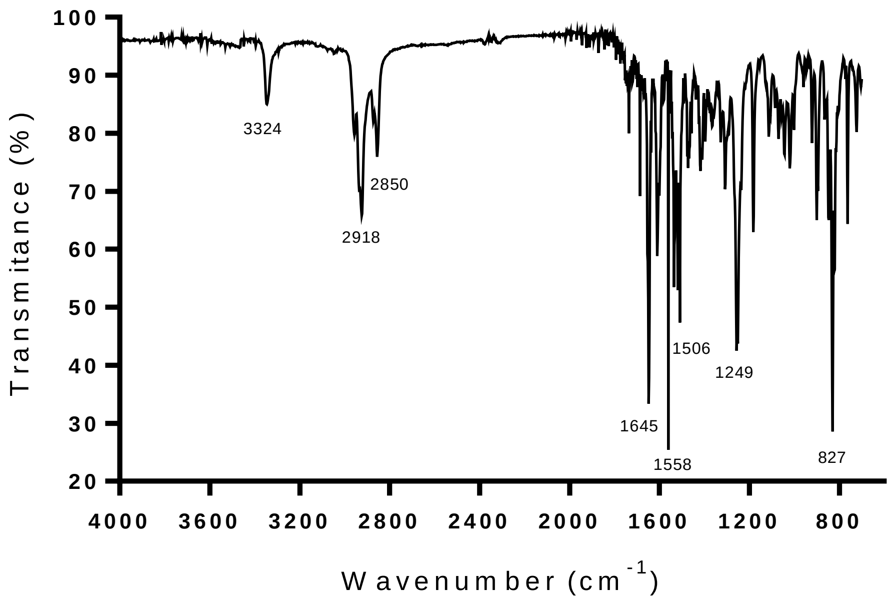

Figure S30 –FT IR spectrum of compound **21**

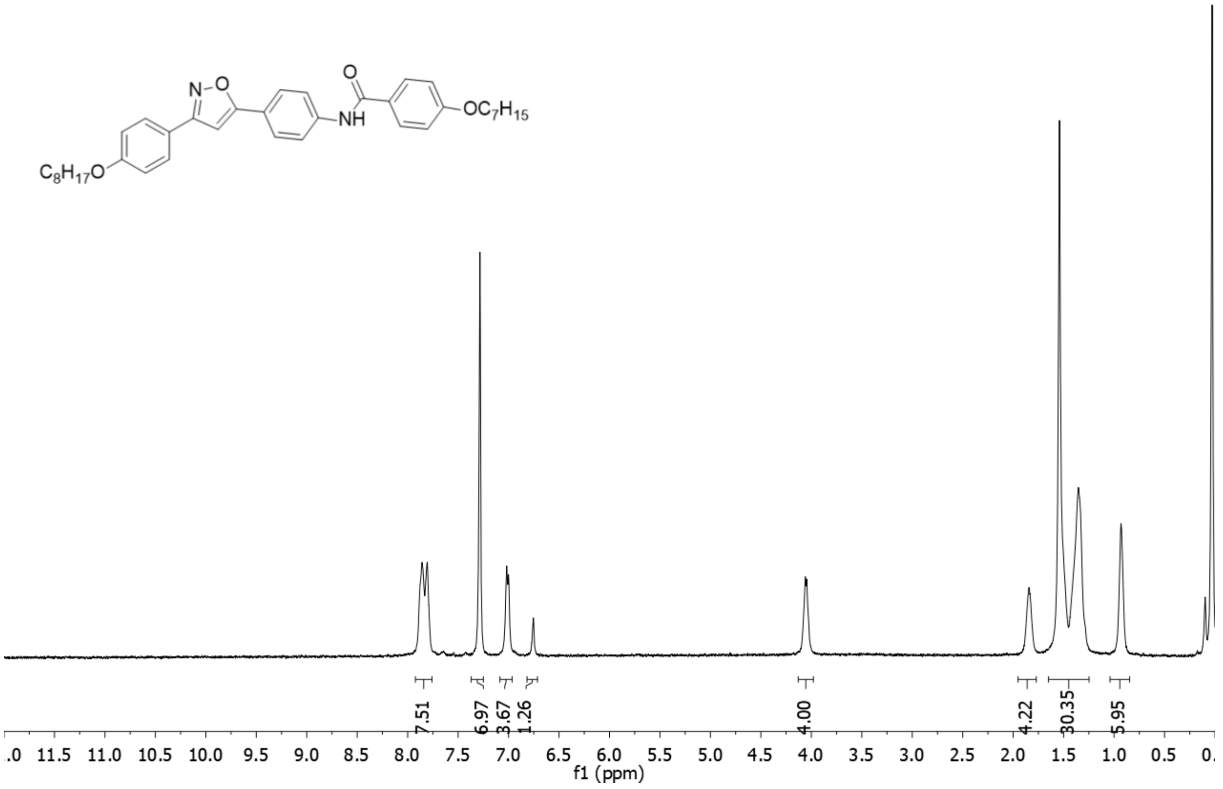

Figure S31 – ^{1}H NMR spectrum of compound **22** ($CDCl_3$, 400 MHz)

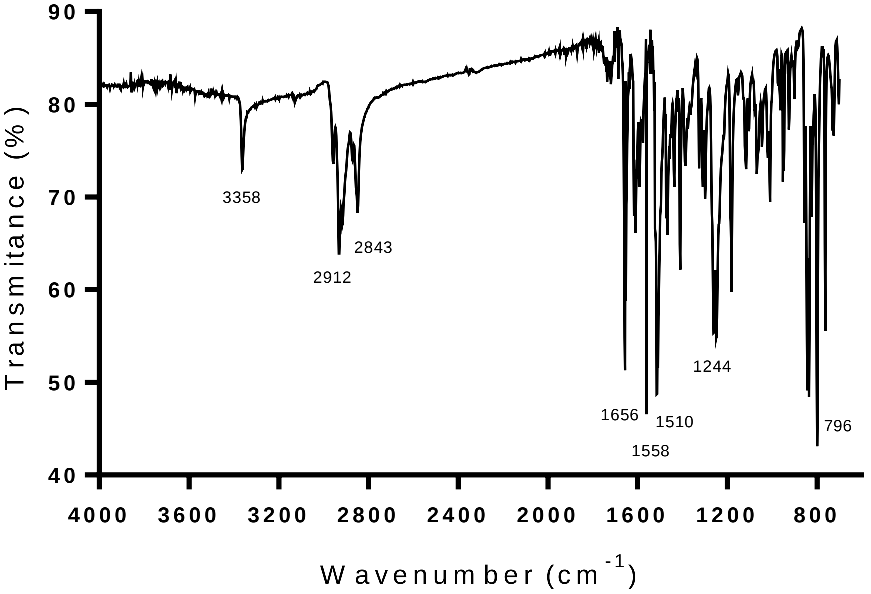

Figure S32 – FT IR spectrum of compound **22**

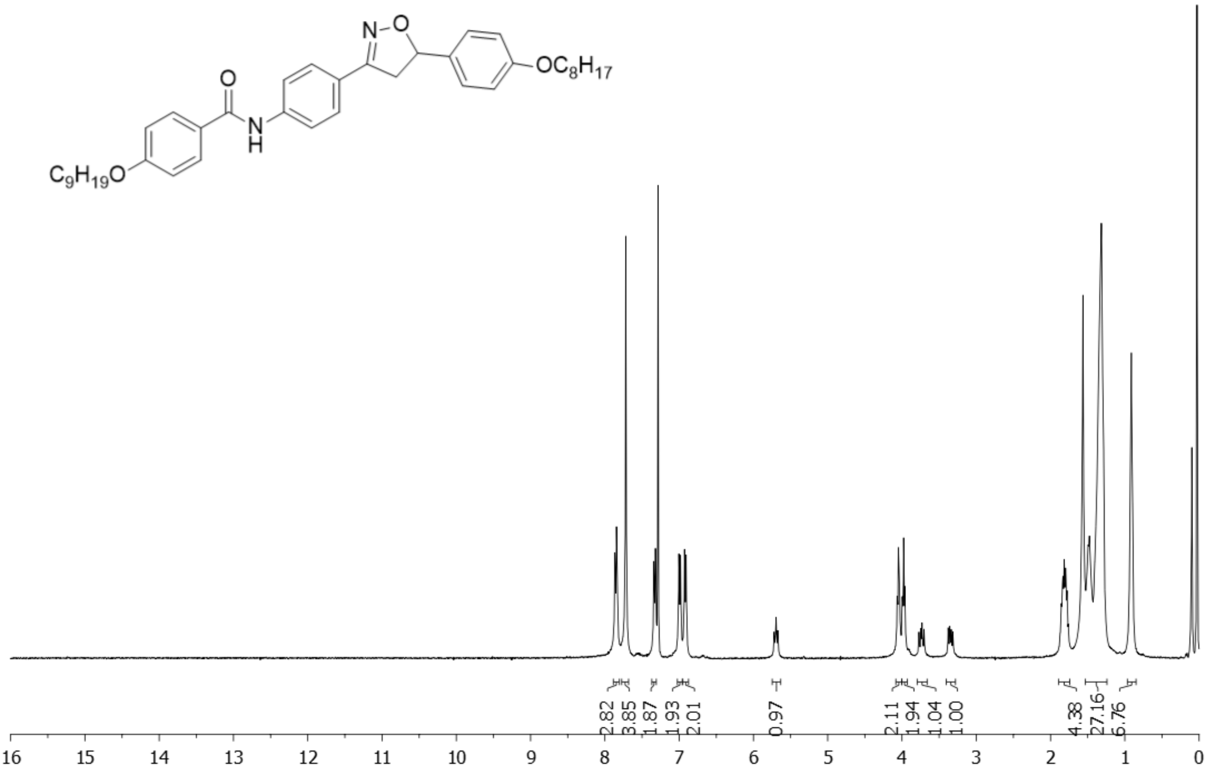

Figure S33 – ^{1}H NMR spectrum of compound **24** ($CDCl_3$, 400 MHz)

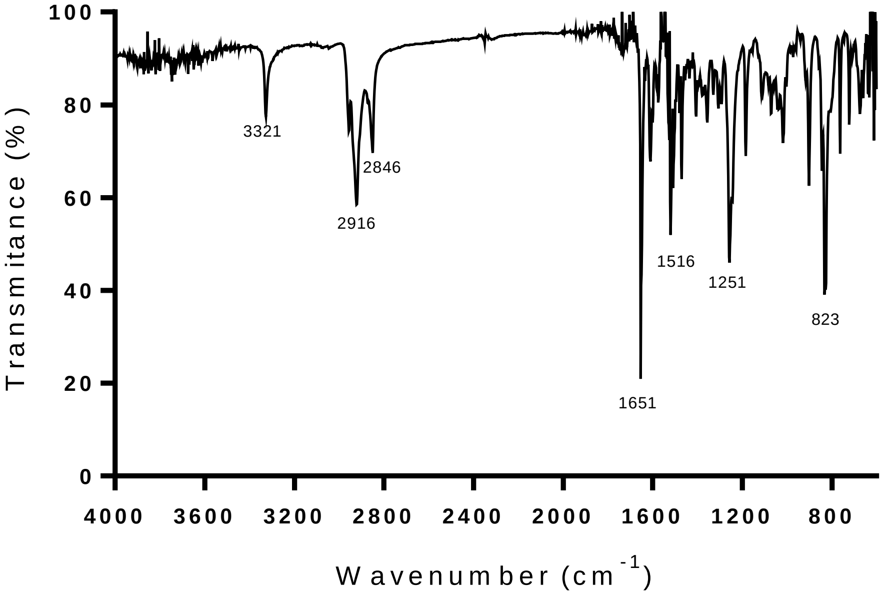

Figure S34 –FT IR spectrum of compound **24**

References

1. Fritsch, L.; Merlo, A. A. *ChemistrySelect* **2016,** *1*, 23-30.
2. da Rosa, R. R., Isoxazolinas e isoxazóis como reais candidatos na preparação de cristais líquidos polares Universidade Federal do Rio Grande do Sul, 2018.
