# Supplementary material for: The use of isoxazoline and isoxazole scaffolding in the design of novel thiourea and amide liquid-crystalline compounds
Source: Beilstein J Org Chem. 2020 Feb 6;16:175–84. doi: 10.3762/bjoc.16.20 (PMC7034240; doi:10.3762/bjoc.16.20)
Supplement: File 2 — Differential scanning calorimetry plots of compounds. [file Beilstein_J_Org_Chem-16-175-s002.pdf]

BEILSTEIN JOURNAL OF ORGANIC CHEMISTRY

Supporting Information

for

The use of isoxazoline and isoxazole scaffolding in the design of novel thiourea and amide liquid-crystalline compounds

Itamar L. Gonçalves, Rafaela R. da Rosa, Vera L. Eifler-Lima and Aloir A. Merlo

Beilstein J. Org. Chem. **2020,** *16,* 175–184. doi:10.3762/bjoc.16.20

Differential scanning calorimetry plots of compounds

License and Terms: This is a supporting information file under the terms of the Creative Commons Attribution License (https://creativecommons.org/licenses/by/4.0). Please note that the reuse, redistribution and reproduction in particular requires that the authors and source are credited.
The license is subject to the *Beilstein Journal of Organic Chemistry* terms and conditions: (https://www.beilstein-journals.org/bjoc)

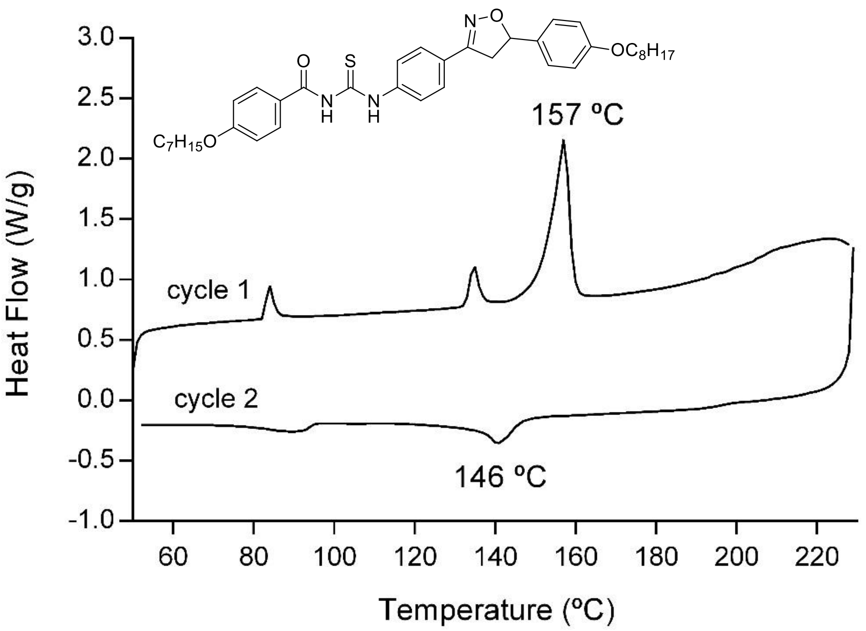

Figure S1 - DSC curve for the thiourea **17a**, upon first heating and cooling cycle at a rate of 10 ºC·min^{-1}.

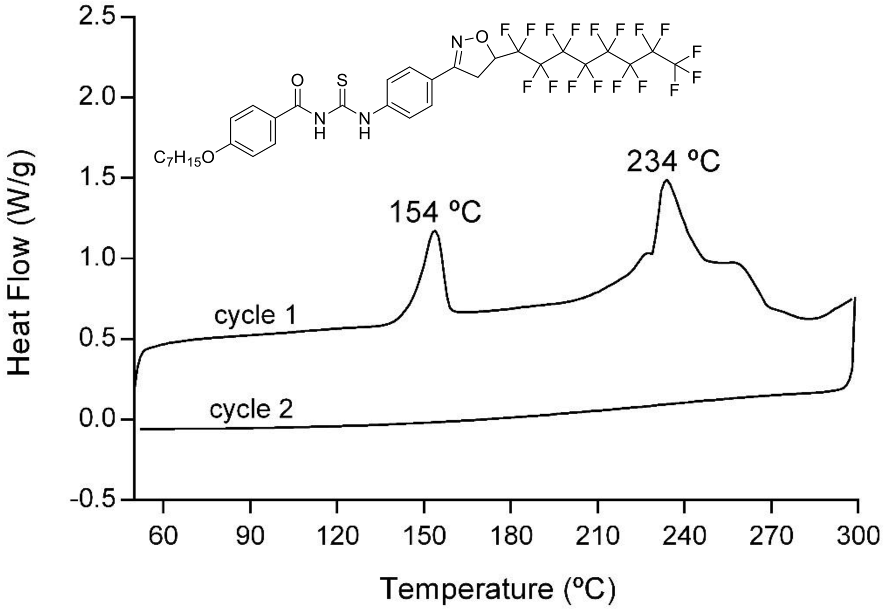

Figure S2 - DSC curve for the thiourea **17b**, upon first heating and cooling cycle at a rate of 10 ºC·min^{-1}.

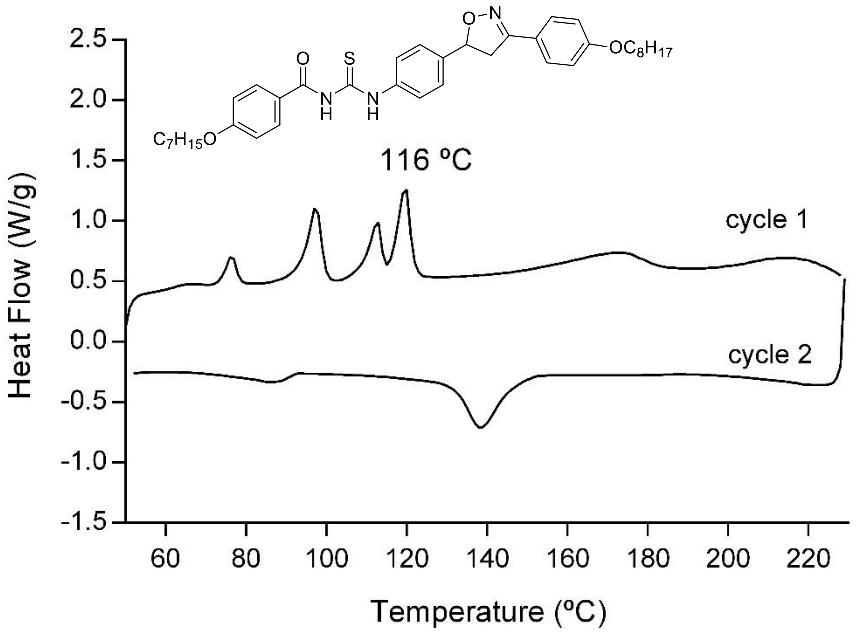

Figure S3 - DSC curve for the thiourea **17c**, upon first heating and cooling cycle at a rate of 10 ºC·min^{-1}.

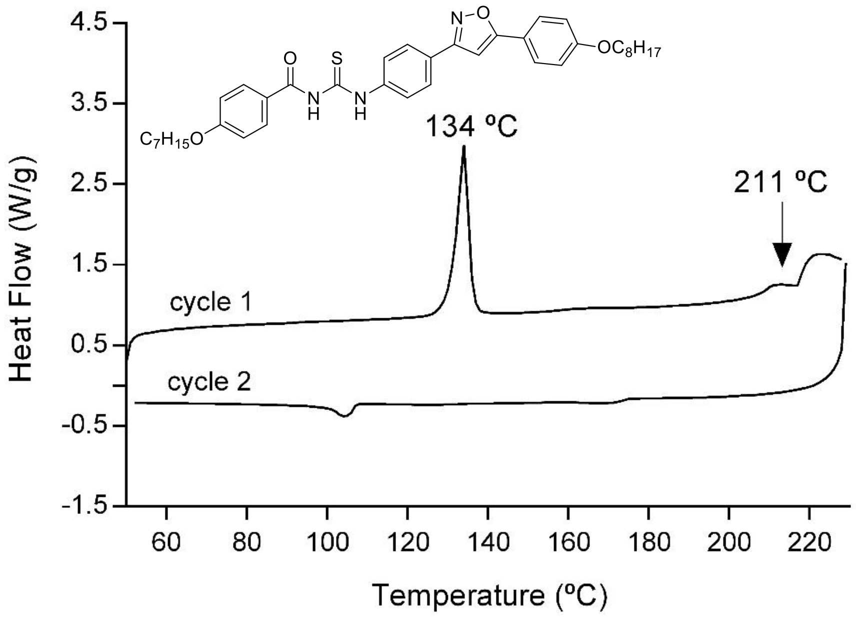

Figure S4 - DSC curve for the thiourea **18a**, upon first heating and cooling cycle at a rate of 10 ºC·min^{-1}.

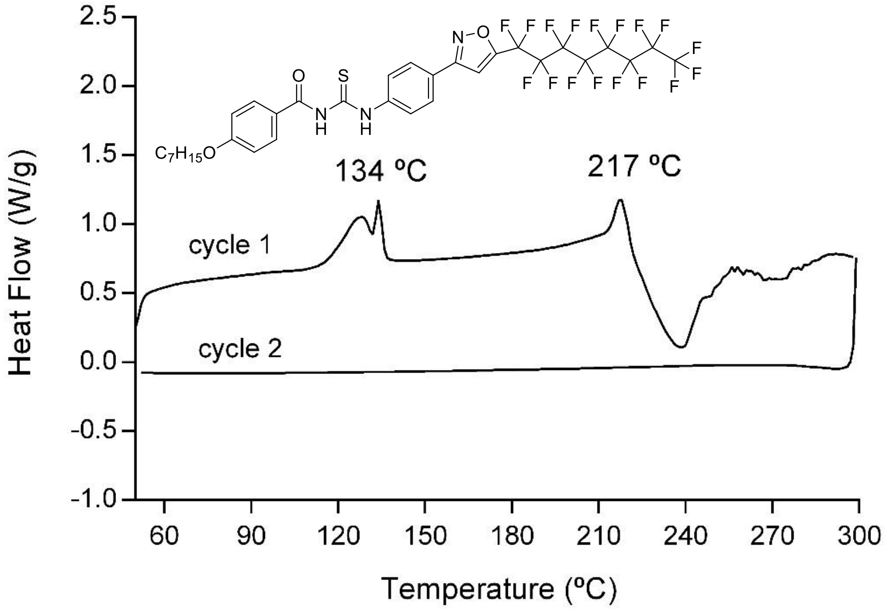

Figure S5 - DSC curve for the thiourea **18b**, upon first heating and cooling cycle at a rate of 10 ºC·min^{-1}.

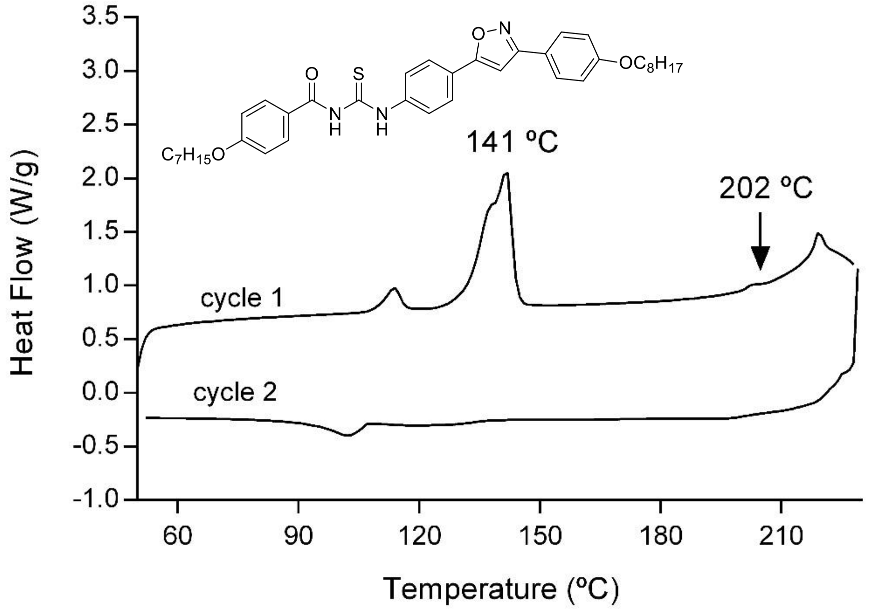

Figure S6 - DSC curve for the thiourea **18c**, upon first heating and cooling cycle at a rate of 10 ºC·min^{-1}.

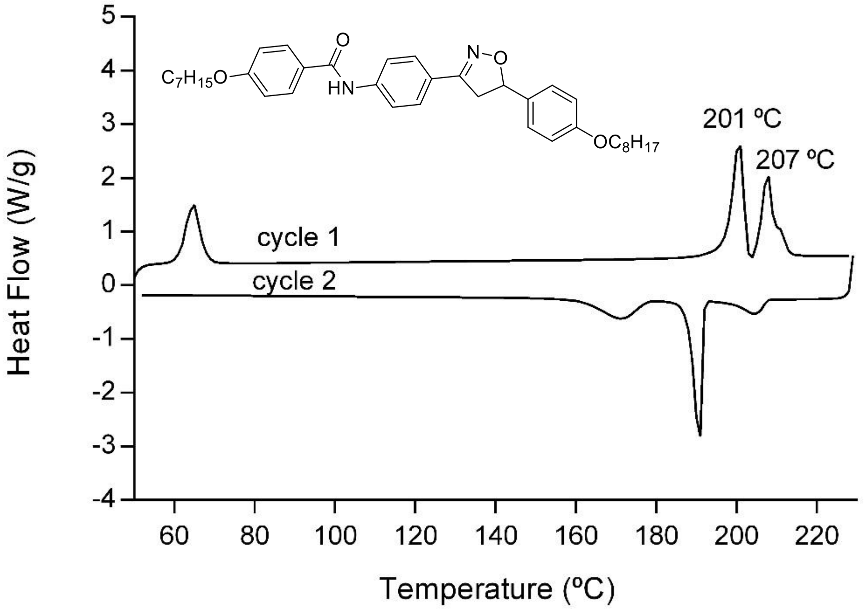

Figure S7 - DSC curve for the amide **19**, upon first heating and cooling cycle at a rate of 10 ºC·min^{-1}.

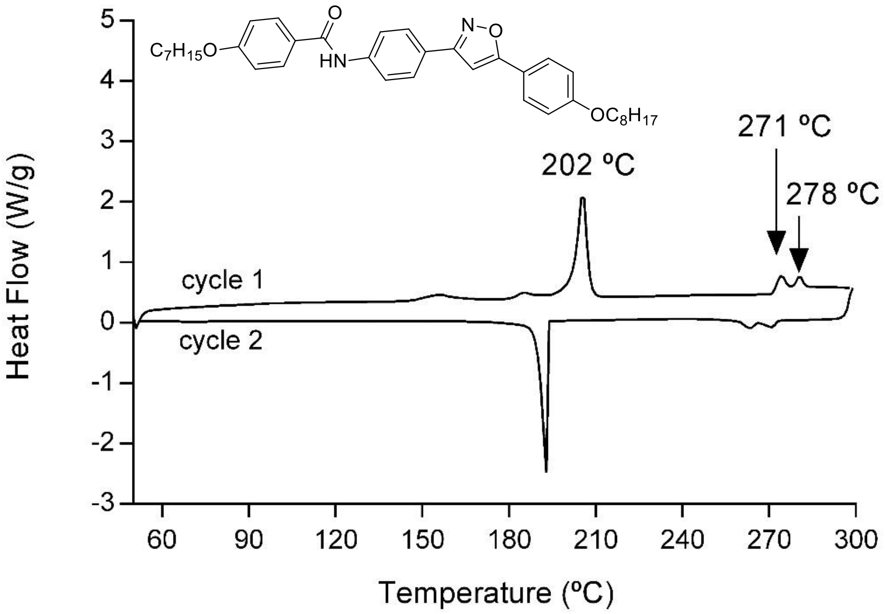

Figure S8 - DSC curve for the amide **20**, upon first heating and cooling cycle at a rate of 10 ºC·min^{-1}.

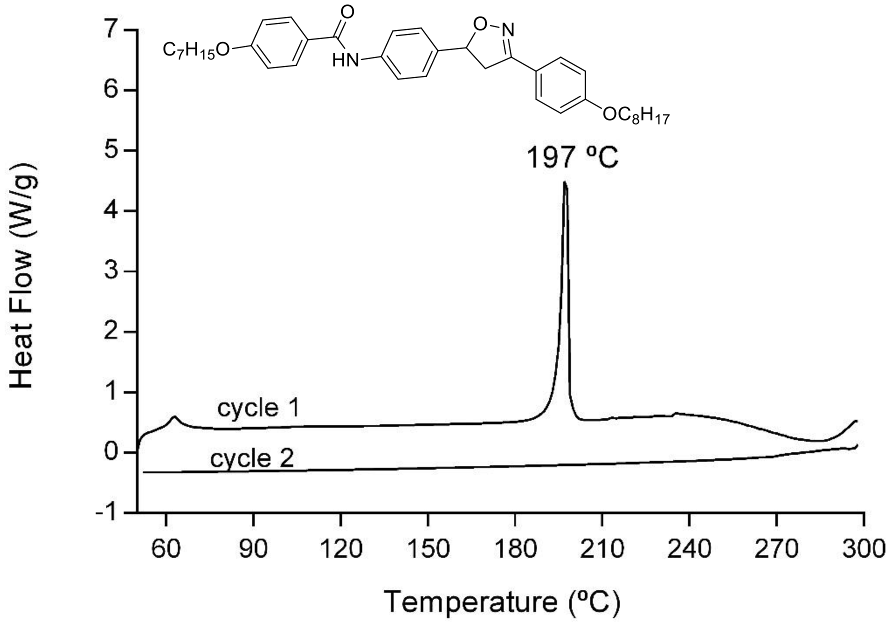

Figure S9 - DSC curve for the amide **21**, upon first heating and cooling cycle at a rate of 10 ºC·min^{-1}.

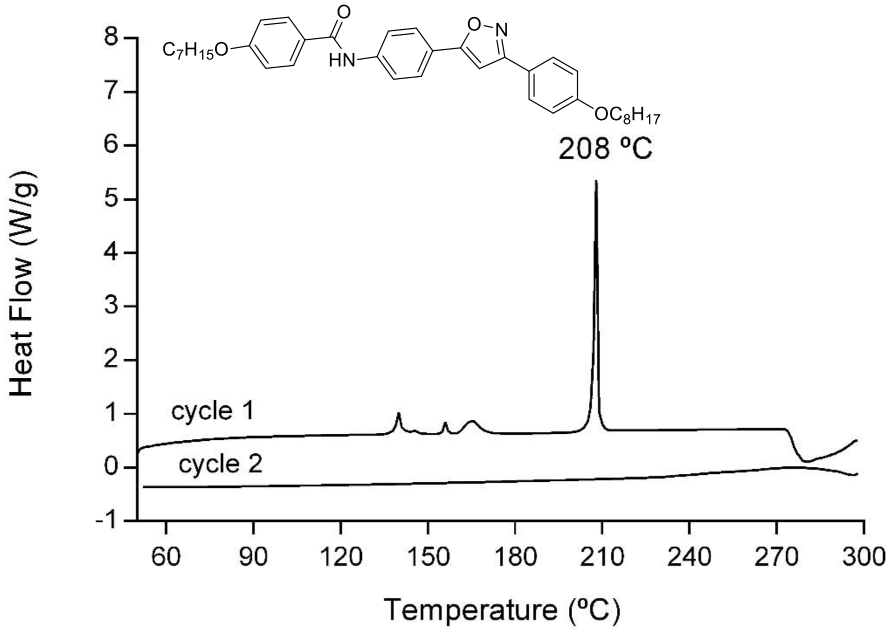

Figure S10 - DSC curve for the amide **22**, upon first heating and cooling cycle at a rate of 10 ºC·min^{-1}.

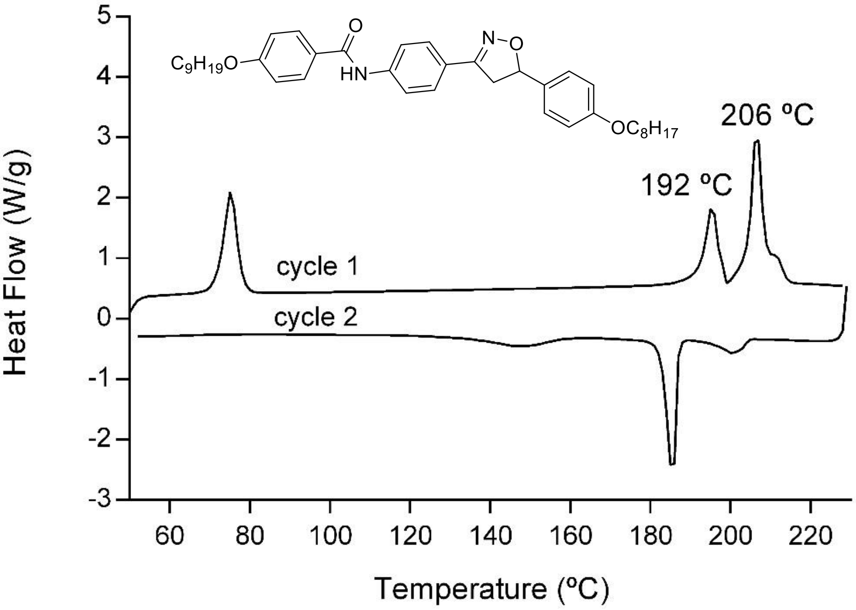

Figure S11 - DSC curve for the amide **24**, upon first heating and cooling cycle at a rate of 10 ºC·min^{-1}.
